# Base excision repair-mediated resistance to cisplatin in KRAS(G12C) mutant NSCLC cells

**DOI:** 10.18632/oncotarget.5019

**Published:** 2015-09-02

**Authors:** Elisa Caiola, Daniela Salles, Roberta Frapolli, Monica Lupi, Giuseppe Rotella, Anna Ronchi, Marina Chiara Garassino, Nikola Mattschas, Stefano Colavecchio, Massimo Broggini, Lisa Wiesmüller, Mirko Marabese

**Affiliations:** ^1^ Laboratory of Molecular Pharmacology, Department of Oncology, IRCCS - Istituto di Ricerche Farmacologiche “Mario Negri”, Milan, Italy; ^2^ Department of Obstetrics and Gynecology of the University of Ulm, Ulm, Germany; ^3^ Laboratory of Cancer Pharmacology, Department of Oncology, IRCCS - Istituto di Ricerche Farmacologiche “Mario Negri”, Milan, Italy; ^4^ Department of Environmental Health Sciences, IRCCS - Istituto di Ricerche Farmacologiche “Mario Negri”, Milan, Italy; ^5^ Centro Nazionale Informazione Tossicologiche, Fondazione Salvatore Maugeri I.R.C.C.S., Pavia, Italy; ^6^ Department of Medical Oncology, Fondazione IRCCS Istituto Nazionale dei Tumori, Milan, Italy

**Keywords:** KRAS, resistance, NSCLC, base excision repair, cisplatin

## Abstract

*KRAS* mutations in NSCLC are supposed to indicate a poor prognosis and poor response to anticancer treatments but this feature lacks a mechanistic basis so far. In tumors, *KRAS* was found to be mutated mostly at codons 12 and 13 and a pool of mutations differing in the base alteration and the amino acid substitution have been described. The different *KRAS* mutations may differently impact on cancerogenesis and drug sensitivity. On this basis, we hypothesized that a different *KRAS* mutational status in NSCLC patients determines a different profile in the tumor response to treatments. In this paper, isogenic NSCLC cell clones expressing mutated forms of *KRAS* were used to determine the response to cisplatin, the main drug used in the clinic against NSCLC. Cells expressing the KRAS(G12C) mutation were found to be less sensitive to treatment both *in vitro* and *in vivo*. Systematic analysis of drug uptake, DNA adduct formation and DNA damage responses implicated in cisplatin adducts removal revealed that the KRAS(G12C) mutation might be particular because it stimulates Base Excision Repair to rapidly remove platinum from DNA even before the formation of cross-links.

The presented results suggest a different pattern of sensitivity/resistance to cisplatin depending on the *KRAS* mutational status and these data might provide proof of principle for further investigations on the role of the KRAS status as a predictor of NSCLC response.

## INTRODUCTION

Lung cancer figures among the leading causes of mortality worldwide [1] and strongly associates with environmental factors and smoking habits [2]. The 5-year prognosis of NSCLC patients is very poor with a percentage of survivors lower than 15% for all stages and lower than 5% for metastatic patients [3]. Only few NSCLC patients, harboring mutations in the *EGFR* gene [4] or presenting the *ALK-EML4* translocation [5], benefit from targeted therapy with erlotinib/gefitinib [4] or crizotinib, [6] respectively. The remaining patients are currently treated with platinum-based combinations [7].

*KRAS* is among the most frequently mutated oncogene in NSCLC and its mutations are present in approximately 20% of lung adenocarcinomas and tumors of smokers [8]. *KRAS* mutations are mainly missense mutations at codon 12 and 13, but rare variants were detected in other codons [9].

*KRAS* is a member of the *RAS* gene family which encodes small G-proteins with intrinsic GTPase activity. GTPase activity leads to protein inactivation and control of downstream effectors involved in multiple pathways including proliferation, differentiation and apoptosis [10]. Point mutations occur in tumors resulting in the loss of intrinsic GTPase activity and consequently in the deregulation of cell proliferation signals and increased aggressiveness of tumors [9–11].

We have shown, at preclinical level, that the the most frequently altered *KRAS* codons in NSCLC have a different response *in vitro* to conventional chemotherapeutic and targeted drugs used in the clinic. In particular the KRAS(G12C) mutation, the most abundant in lung cancer, associates with a weaker response to cisplatin treatment compared to wt and other tested mutations [12]. We have recently shown in a prospective study that NSCLC patients with mutated *KRAS* tumor had a worse response to first-line platinum-based treatment compared to KRAS(wt) patients [13] and unpublished results.

In the clinic, *KRAS* mutated patients so far cannot benefit from any targeted therapy and are treated in first-line with platinum based compounds as the KRAS(wt) patients. In this paper we characterized the role of *KRAS* mutations at position 12, in particular the KRAS(G12C) mutation, in mediating response to cisplatin treatment with the aim to elucidate the mechanisms of cisplatin resistance induced by this mutation and to give the rationale of possible new pilot clinical trials aimed at stratifying patients on the basis of *KRAS* mutations.

## RESULTS

### *In vitro* cisplatin response as a function of the KRAS status

**Figure 1:**
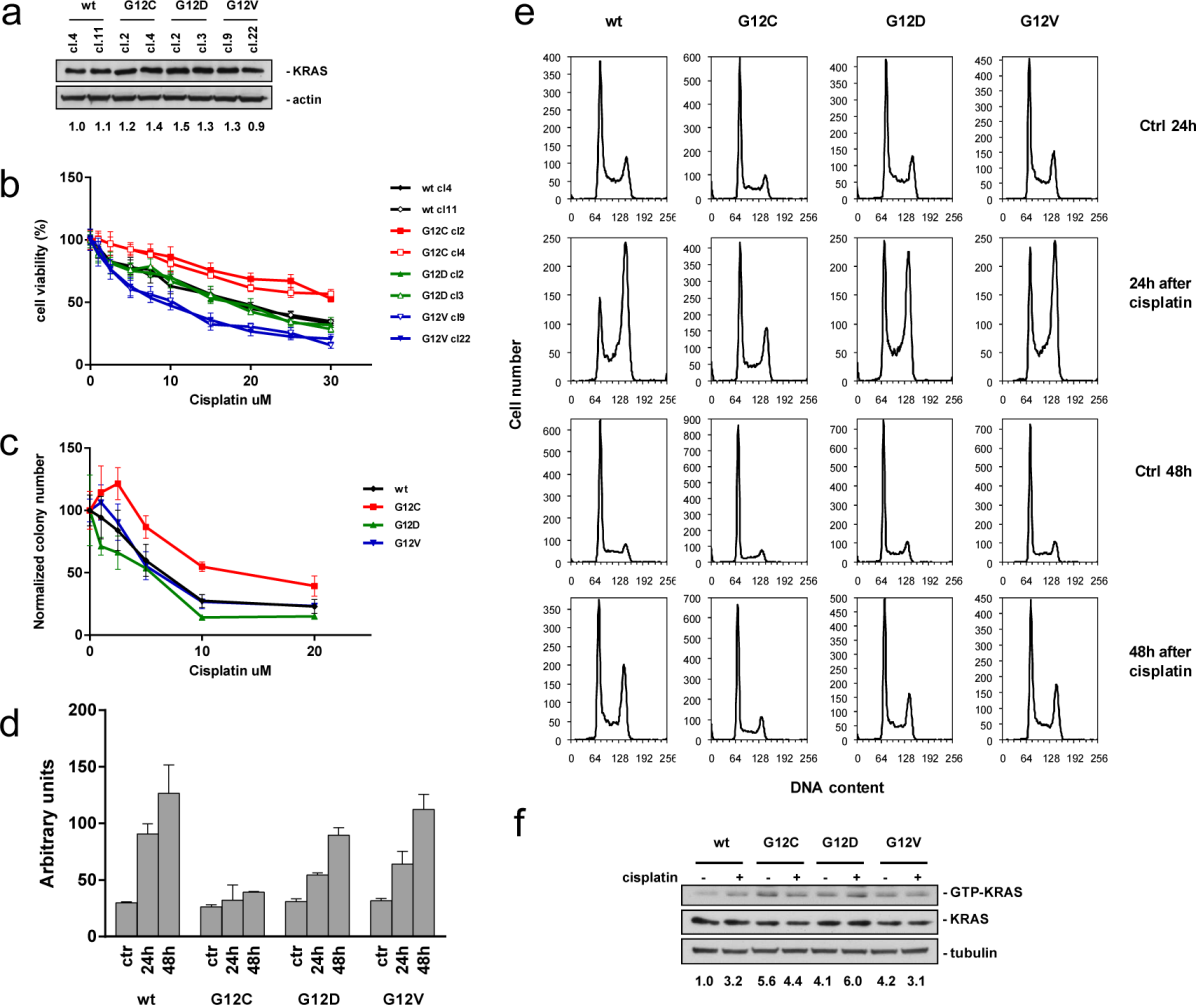
Characterization of KRAS expressing H1299 tumor cells **a.** Representative Western blot analysis demonstrating comparable expression of exogenous KRAS variants in the isolated clones. Actin was used as loading control. Values reported below the Western blot represent protein band intensities normalized with Actin. Protein levels of KRAS(wt) clone (cl.) 4 were set to 1. Two independent experiments were performed. **b.** Response of cells to cisplatin detected by MTS assay. The average of 3 independent experiments and SD are shown. Statistical analysis results are reported in Supplementary Table S1. **c.** Colony numbers plotted as percentages of untreated controls. The average of 4 different biological replicates and SD are shown. Statistical analysis results are reported in Supplementary Table S1. **d.** Caspase 3 and 7 activities assessed by the Caspase-Glo 3/7 Assay 24 h and 48 h after recovery. The average of 3 different biological replicates and SD are shown. Statistical analysis results are reported in Supplementary Table S1. **e.** Cell cycle phase distribution. Percentages are listed in Supplementary Table S2. **f.** Levels of GTP-KRAS assessed by pull down with the recombinant RAS-binding domain of RAF and detected by anti-KRAS antibody. Total lysates were also immunoblotted with anti-KRAS and anti-Tubulin antibody as loading control. Values reported below the Western blot represent protein band intensities normalized with Tubulin band intensities. Protein levels of untreated KRAS(wt) clone were set to 1.

Using isogenic NCI-H1299 derived clones, expressing comparable KRAS protein levels (Figure 1a), we determined the activity of cisplatin *in vitro* by using two independent clones for each KRAS variant. As already reported for one set of clones and different chemotherapeutic agents in our previous manuscript [12], the expression of a specific KRAS mutant induced a distinct sensitivity pattern detected by MTS assay. Both clones expressing the KRAS(G12C) showed a weaker response to cisplatin compared to KRAS(wt), KRAS(G12D) or KRAS(G12V) clones (Figure 1b).

Statistical analysis did not detect differences between independent clones harboring the same mutation, but between clones expressing different KRAS variants. Assessment of IC50 values from the mean curves of two clones expressing the same KRAS variant indicated an approximately two-fold difference between KRAS(wt) (IC50 = 16.32 ± 2.78 uM) or KRAS(G12D) (IC50 = 17.53 ± 1.99 uM) and KRAS(G12C) clones (IC50 > 30 uM) and even a three-fold difference between KRAS(G12C) and KRAS(G12V) clones (IC50 = 12.92 ± 3.21 uM).

This finding was strengthened by clonogenicity testing, revealing reduced sensitivity of the KRAS(G12C) clone to cisplatin compared to the other clones (Figure 1c). The reduced activity of cisplatin in KRAS(G12C) expressing cells was further confirmed in other isogenic systems expressing the different KRAS mutants and in NSCLC cells with a different *KRAS* status (Supplementary Figure S5).

We then performed a series of experiments aimed at understanding the reason for the different response to cisplatin of KRAS(G12C) clones. Cisplatin did not induce the central step of apoptosis, namely caspase 3/7 cleavage, in the KRAS(G12C) clone, however, in KRAS(wt) and to a slightly lesser extent also in KRAS(G12D) and KRAS(G12V) clones the drug stimulated the activation of caspase 3/7 (Figure 1d).

To understand whether differences in response of KRAS(G12C) cells were associated with changes in cell cycle perturbations, we treated the clones with cisplatin and performed flow cytometric DNA content analysis. KRAS(wt), KRAS(G12D) and KRAS(G12V) clones showed an at least two-fold accumulation of cells in G2/M phase of the cell cycle 24 h after treatment, which was not observed in the less cisplatin-sensitive KRAS(G12C) clone. Forty-eight hours after treatment, KRAS(wt), KRAS(G12D) and KRAS(G12V) clones showed a partial re-distribution of G2/M phase cells in the different phases (Figure 1e, Supplementary Table S2). In KRAS(G12C) cells a G2/M phase accumulation was not even observed at this later time-point.

To exclude the possibility that different drug responses in the clones are due to differences in KRAS activation, guanosine-5′-triphosphate-bound KRAS (GTP-KRAS) levels were assessed before and after cisplatin treatment. At basal level, mutated KRAS harboring clones showed elevated levels of GTP-KRAS compared to the KRAS(wt) clone (Figure 1f). Upon cisplatin treatment, mutant clones did not accumulate activated KRAS whereas the GTP-KRAS in KRAS(wt) clone increased and reached levels similar to KRAS mutated clones.

### *In vivo* cisplatin response of KRAS(G12C) expressing NSCLC cells

**Figure 2:**
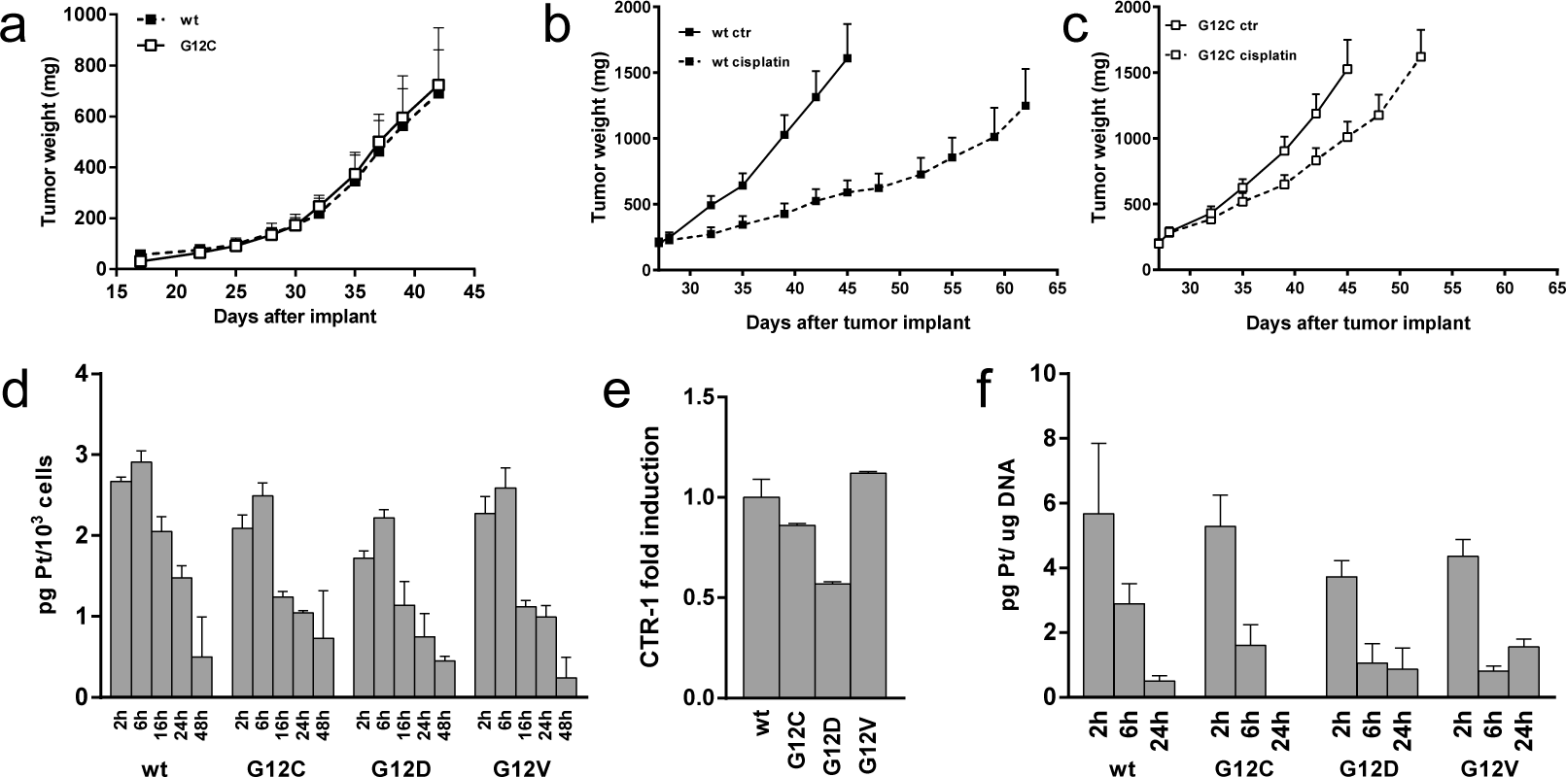
Cisplatin *in vivo* response and platinum intracellular levels **a.** Tumor growth after injection of KRAS(G12C) or KRAS(wt) cells in opposite sides of nude mice (*N* = 5). Statistical analysis was performed using two-way ANOVA test and Bonferroni post-test for multiple comparisons and no differences were detected. **b–c.** Tumor growth inhibition activity on KRAS(wt) (b) or KRAS(G12C) (c) clones injected mice (*N* = 8) treated with cisplatin intravenously at 5 mg/Kg 3 times every 7 days or vehicle. Means and SEM are shown. Statistical analysis results are reported in Supplementary Table S1. **d.** Assessment of intracellular platinum concentration. The average of 3 different biological replicates and SD are shown. Statistical analysis results are reported in Supplementary Table S1. **e.** Relative expression levels of the CTR-1 measured by real time PCR at basal level. KRAS(wt) clone was set to 1. The average of 3 different technical replicates and SD are shown. **f.** Assessment of platinum adducts bound to DNA after cisplatin treatment for 2 h at 10 uM. The average of 3 different biological replicates and SD are shown. Statistical analysis results are reported in Supplementary Table S1.

To examine whether the particular cisplatin response of KRAS(G12C) cells observed *in vitro* was maintained *in vivo*, KRAS(G12C) cl.2 and KRAS(wt) cl.4 were subjected to xenotransplantation experiments. We injected the KRAS(G12C) and the KRAS(wt) expressing clones in contralateral positions of the same mice. Corresponding tumor growth rates were indistinguishable, enabling comparison of cisplatin antitumor activity in the two clones *in vivo* (Figure 2a). Once unilaterally injected tumors reached approximately 200 mm^3^ in size, mice were randomized and treated with cisplatin. Following this treatment, KRAS(wt) clone showed a tumor weight reduction between treated and control groups reaching statistical significance on days 39, 42 and 45 (*p* < 0.0001) after tumor implant with a best treated over control ratio (T/C) of 36% at day 45 (Figure 2b). The KRAS(G12C) expressing clone showed a best T/C of only 66% at day 45 (Figure 2c). Only in the cisplatin-treated group with KRAS(wt) tumors, all mice (8/8) reached day 62. In the KRAS(G12C) group, all mice had to be sacrificed at day 52 because tumors reached the maximum tumor volume compatible with animal health status (10% of the body weight). In conclusion, KRAS(G12C) tumors showed a reduced response to cisplatin compared to KRAS(wt) tumors *in vivo*, confirming the results obtained *in vitro*.

### Evaluation of MAPK and PI3K signaling downstream of KRAS

To further delineate KRAS activation in the clones, activation of PI3K and MAPK pathways was evaluated at different time-points after cisplatin treatment (Supplementary Figure S1a). No major cisplatin-induced accumulation of p-MEK above MEK signals was detectable. Cisplatin induced a transient increase of p-Erk peaking around 2–16 h post-treatment in all the clones. A lower extent of p-Erk was detected at later time-points than 2 h post-treatment in the KRAS(G12V) clone when compared with the others.

Analysis of p-Akt(thr308), p-S6(ser235/236) and p-4EBP1(thr37/46) did not reveal clear cisplatin-induced changes in the different clones. The pattern of p-p70S6K(thr389) was similar in all clones: p70S6K was activated 2 h after treatment and inactivated after 48 h. KRAS(wt), KRAS(G12D) or KRAS(G12V) clones showed accumulation of p-PRAS40(thr246) starting around 16–24 h after cisplatin treatment. The KRAS(G12C) clone showed a p-PRAS40 already before treatment and no further accumulation of the p-PRAS40 form after 16 h post-treatment. Altogether, kinase signaling at least in response to cisplatin treatment was comparable in all the clones.

### Intracellular amount of cisplatin

As graphically shown in Figure 2d no differences in the intracellular content of cisplatin were found at least between the mutant expressing KRAS clones at any time-point of the experiment. Expression of the copper influx transporter CTR-1, that is also a major influx transporter for cisplatin, was correlated to resistance of this drug [14]. Its expression, assessed by real-time PCR, was similar in the clones (Figure 2e) with a modest, not significant, lower expression in the KRAS(G12D) clone.

The GST activity and the intracellular GSH amount were determined in untreated cells but did not reveal significant differences. In agreement, equivalent responses were found in the clones following treatment with the alkylating agent melphalan, whose resistance is dependent on the GST/GSH levels [15] (Supplementary Figure S1b).

To further investigate whether cisplatin similarly reached its target in the cells, platinum bound to DNA was estimated by DRC-ICP-MS. Maximum levels of platinum bound to DNA were measured 2 h after treatment and were similar in the different clones. On the contrary, 24 h post-treatment, the level of platinum bound to DNA was below the detection limit in the KRAS(G12C) clone, while it was still measurable in all the other clones (Figure 2f). Altogether these data excluded impaired uptake, faster export or reduced DNA adduct formation of cisplatin despite rapid adduct disappearance in KRAS(G12C) cells.

### Role of DNA damage signaling in differential cisplatin response

**Figure 3:**
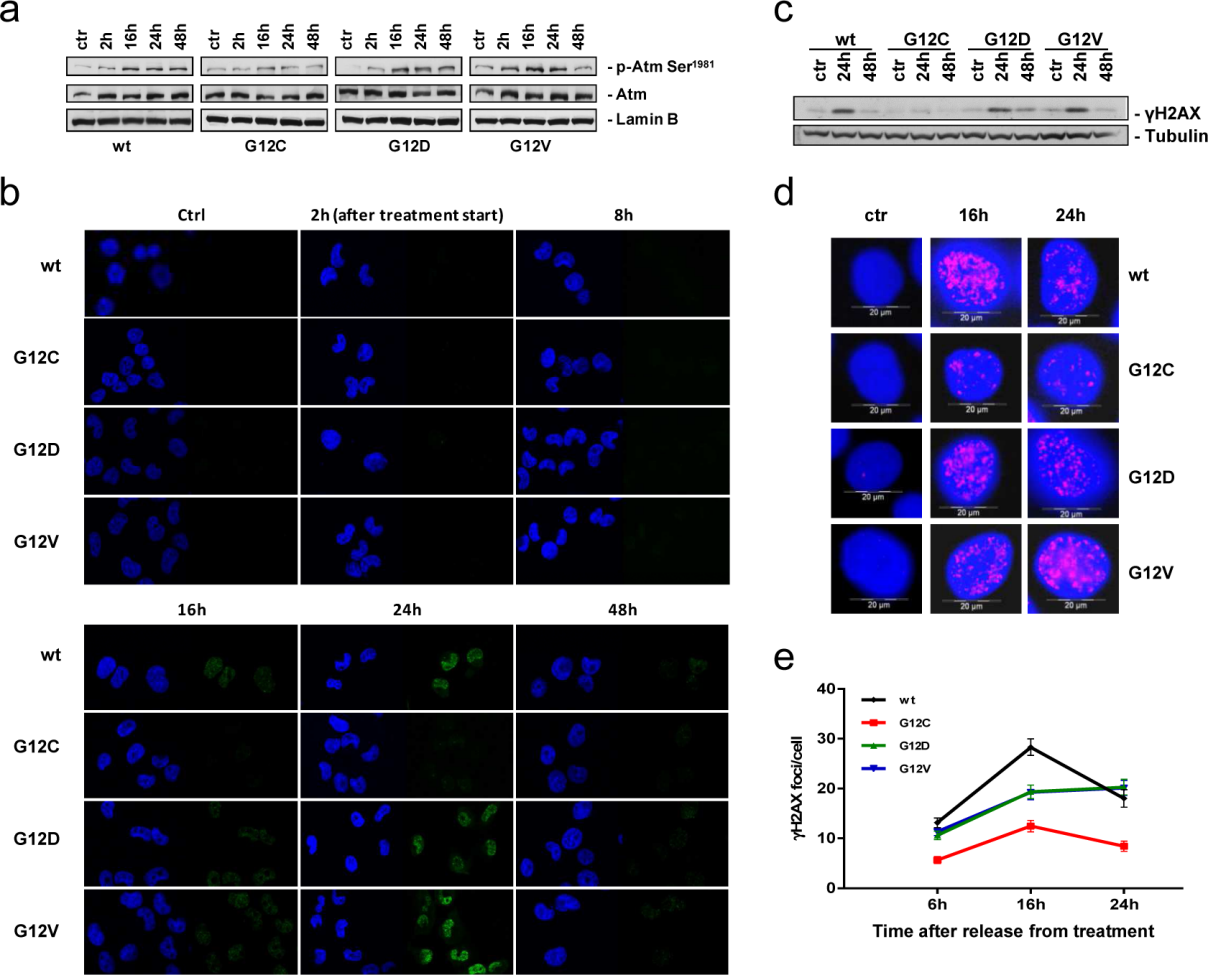
DNA damage response after cisplatin treatment **a.** Representative Western blot analysis reporting the expression and phosphorylation of ATM on serine 1981 in cells treated or not with cisplatin. Lamin B was used as loading control. Graphical presentation of p-Atm Ser^1981^ levels from 2 experiments is shown in Supplementary Figure S1c. **b.** Phosphorylation of H2AX histone (γH2AX, green) detected by immunofluorescence after release from cisplatin treatment. DAPI (blue) was used to counterstain the nuclei. Scale bar: 25 um. **c.** Representative Western blot analysis visualizing phosphorylation of H2AX after release from cisplatin treatment. Tubulin was used as loading control. Two independent experiments have been performed. **d.** Representative images for γH2AX foci quantification. **e.** γH2AX immunolabeled foci from 2–4 slides and 2 independent experiments were scored by automated quantification in 50 nuclei per slide and graphically presented. Mean values and SEM are shown. Statistical analysis results are reported in Supplementary Table S1.

We then wished to investigate whether cisplatin-induced DNA damage-signaling differed among KRAS expressing clones by assessing ATM, the best known apical activator in response to DNA DSBs [16]. We analyzed ATM activation by Western blot detection of the phosphorylated form (p-ATM) of the protein. A 4–5-fold maximum activation was detected in KRAS(wt), KRAS(G12D) and KRAS(G12V) clones 16–24 h after treatment. The less sensitive KRAS(G12C) clone showed only a two-fold increase in the p-ATM post-treatment (Figure 3a).

To understand if differential ATM activation resulted in a different regulation of downstream proteins, the kinetics of γH2AX foci formation and disappearance in the course of DNA repair after cisplatin treatment were investigated at defined time-points. The time course revealed appearance of γH2AX in KRAS(wt), KRAS(G12D) and KRAS(G12V) clones 16 h after treatment, maintenance until 24 h and decline during the subsequent 24 h. In the KRAS(G12C) clone γH2AX signals were almost undetectable during the whole experiment (Figure 3b). Western blot analysis of γH2AX after treatment confirmed the immunofluorescence analysis (Figure 3c). Quantification of distinct nuclear γH2AX foci applying a scaled-up cisplatin dose revealed a statistically significant reduction of foci scores 6 h, 16 h, and 24 h after exposure in KRAS(G12C) cells compared with the other clones (Figure 3d, 3e).

To exclude that KRAS(G12C) expressing cells presented some defects in DNA damage detection, γH2AX was assessed both 24 h after high equitoxic cisplatin doses and at different time-points after IR treatment that directly induces DNA DSBs. Using equitoxic concentrations of platinum resulted in comparable γH2AX levels in the clones (Supplementary Figure S2). Furthermore, all the clones treated with 5 or 7.5 Gy X-ray displayed marked γH2AX signals at both doses and at all the time-points (90 min, 6 h, 24 h) of the experiment (Supplementary Figure S3a, S3b). In agreement, viability after X-ray was similar among clones (Supplementary Figure S3c).

### Analysis of NER and DSB repair mechanisms in mutant KRAS expressing cells

**Figure 4:**
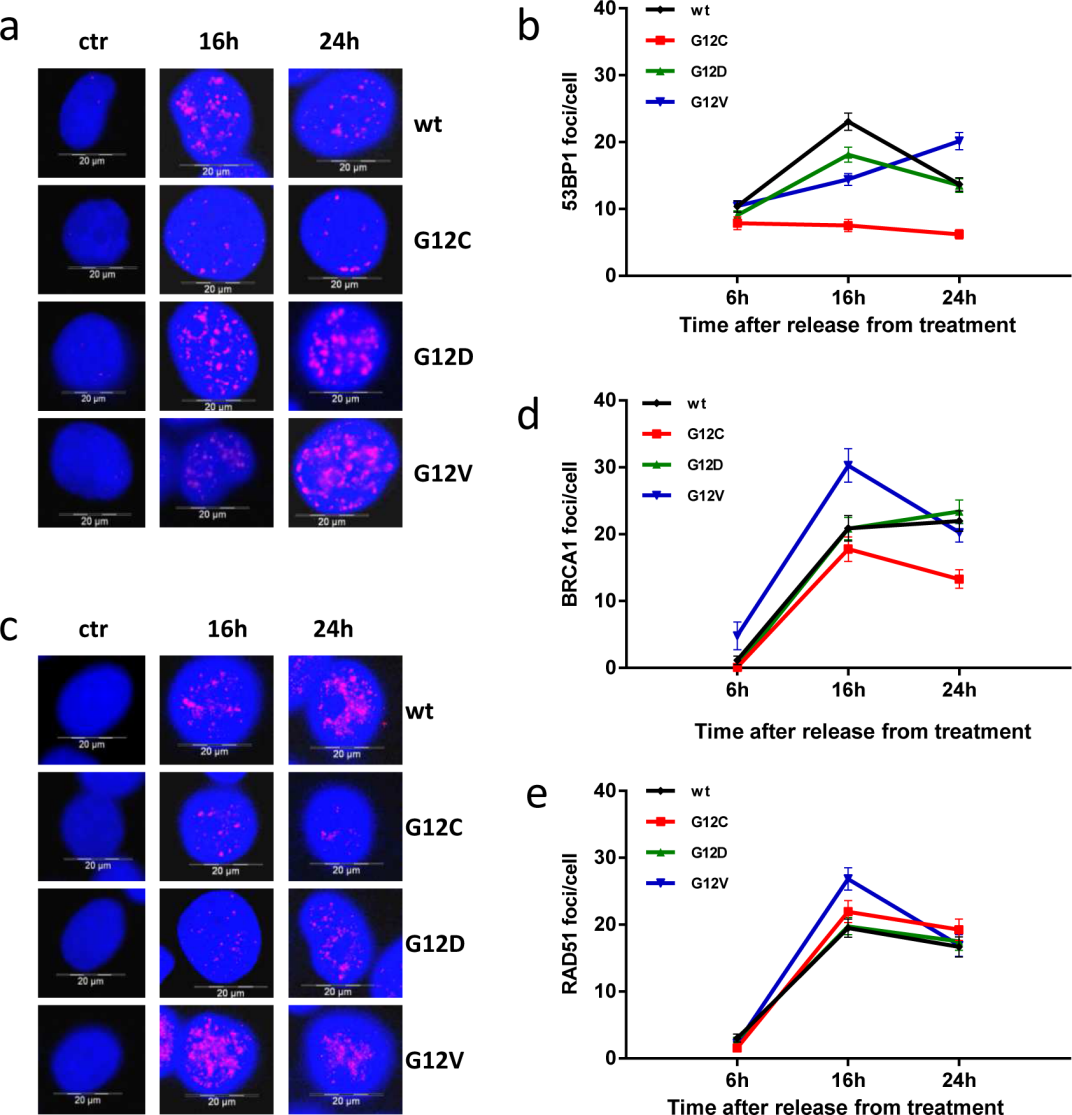
Focal accumulation of DSB repair proteins after cisplatin treatment **a** and **c.** Representative images for 53BP1 (a) and BRCA1 (c) foci quantification after treatment with cisplatin were obtained as for γH2AX in Figure 3d. **b, d** and **e.** 53BP1 (b), BRCA1 (d) and RAD51 (e) foci were quantified and graphically presented as for γH2AX in Figure 3e. Mean values and SEM are shown. Statistical analysis results are reported in Supplementary Table S1.

DNA crosslink repair following cisplatin treatment was previously reported to involve NER mediated incisions and repair of resulting DSB intermediates by HR [17]. To understand the molecular cause underlying diminished accumulation of the DNA damage marker γH2AX in KRAS(G12C) cells, we dissected DSB repair components by immunofluorescence microscopic analysis. When monitoring nuclear 53BP1 and BRCA1 foci, two antagonistic components involved in pathway choice decisions between NHEJ and HR [18], we found reduced foci numbers of 53BP1 16 h and 24 h and of BRCA1 24 h post-treatment in KRAS(G12C) cells (Figure 4a–4d). Analysis of the HR recombinase RAD51 did not reveal statistically significant differences in foci numbers (Figure 4e). Given these results, NER and pathway-specific DSB repair activities were evaluated to examine their potential involvement in the peculiar response of KRAS(G12C) clone to cisplatin.

**Figure 5:**
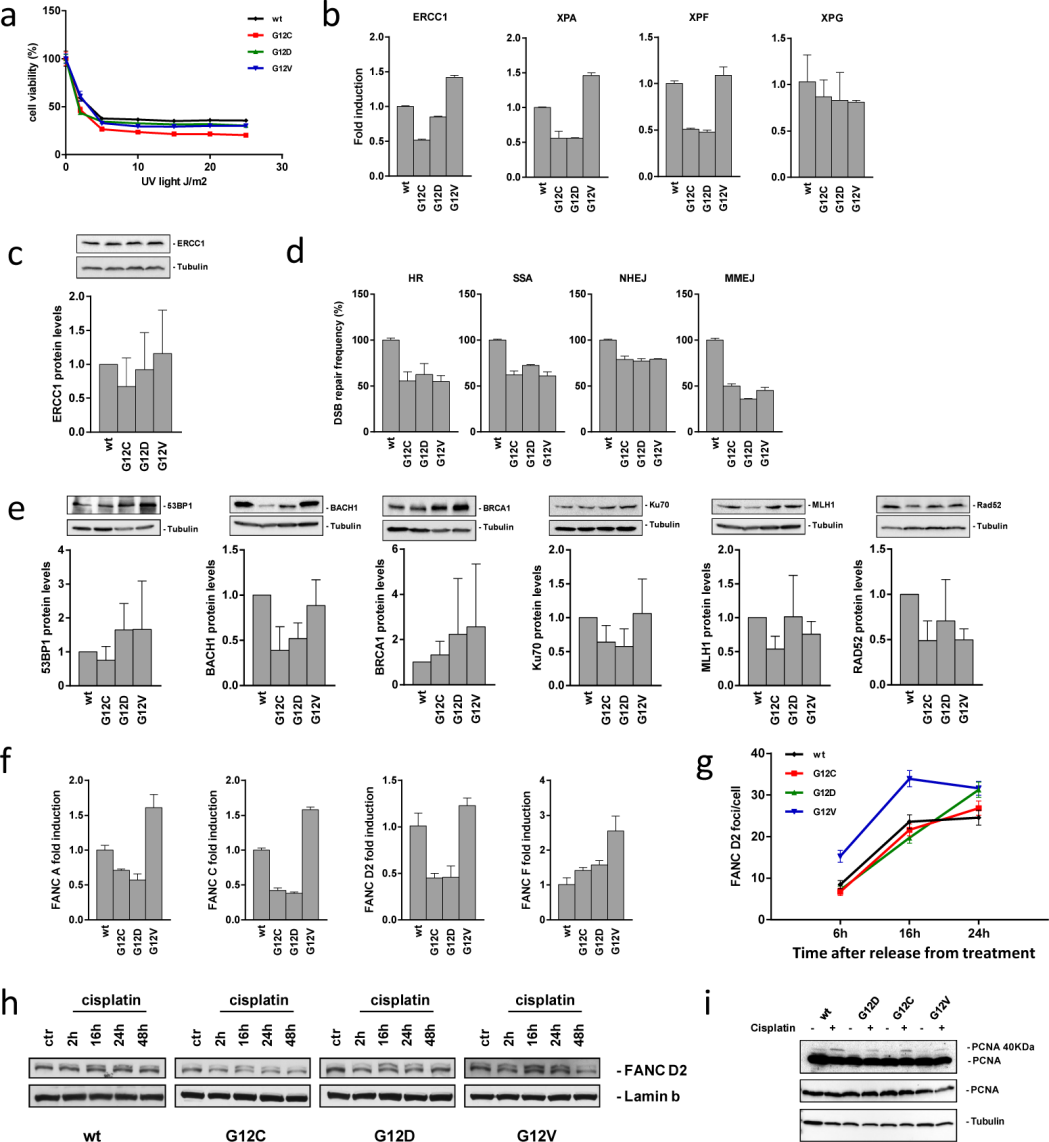
DSB repair activities and analysis of alternative DNA repair pathways **a.** Response of cells to treatment with UV light detected by MTS assay. The data of the survival curves were plotted as percentages of untreated controls 72 h after irradiation. The average of 3 different experiments and SD are shown. Statistical analysis results are reported in Supplementary Table S1. **b.** Relative mRNA expression levels of genes involved in NER measured by real time PCR at basal level. Values for the KRAS(wt) clone were set to 1. The average of 3 different technical replicates and SD are shown. **c.** Expression of endogenous ERCC1 protein in the clones and its graphical presentation. Band intensities of Tubulin were used for individual normalization and values for the KRAS(wt) clone were set to 1. Quantification from 4 independent Western blots (means, SD) are shown. Statistical analysis was performed using one-way ANOVA test and Bonferroni post-test for multiple comparisons and no differences were detected. **d.** DSB repair frequencies. Values for cells from the KRAS(wt) expressing clone were set to 100% (absolute values for HR: 16%; SSA: 31%; NHEJ: 44%; MMEJ: 5%) and relative mean frequencies and SEM from 6 measurements are shown. Statistical analysis results are reported in Supplementary Table S1. **e.** Endogenous 53BP1, BACH1, BRCA1, KU70, MLH1 and RAD52 protein levels in the clones and their quantification. Band intensities of the Tubulin were used for individual normalization. Quantification from 2–6 independent Western blots (means, SD) are shown. Statistical analysis results are reported in Supplementary Table S1. **f.** Relative mRNA expression levels of genes involved in the FA repair pathway measured by real time PCR in the clones at basal level. Values for the KRAS(wt) clone were set to 1. The average of 3 different technical replicates and SD are shown. **g.** FANCD2 immunolabeled foci from 2 slides from independent experiments were scored by automated quantification in 50 nuclei per slide and graphically represented. Mean values and SEM are shown. Statistical analysis results are reported in Supplementary Table S1. **h.** Representative Western blot analysis displaying levels of ubiquitylated, i.e. activated FANCD2 protein (upper band) and unmodified FANCD2 (lower band) in the clones treated or not with cisplatin. Lamin B was used as loading control. Two independent experiments have been performed. **i.** Representative Western blot analysis showing levels of mono-ubiquitylated, i.e. activated PCNA protein (long exposure, upper band) and the unmodified form of the protein (long exposure, lower band, and short exposure) in the clones treated or not with cisplatin. Tubulin was used as loading control.

Functionality of the NER system was indirectly evaluated by UV radiation treatment generating DNA lesions which are mainly repaired through NER [19]. Similar responses in the different clones were observed with a higher sensitivity rather than resistance of KRAS(G12C) cells (Figure 5a). We also analyzed relative mRNA expression of the NER genes *ERCC1*, *XPA*, *XPF* and *XPG* in the clones. Some differences were detected, however, they did not appear to be associated with the differential responses of the clones to treatment (Figure 5b). A trend to reduced *XPA* and *XPF* expression was observed in KRAS(G12C) but also in KRAS(G12D) cells. *ERCC1* mRNA was low in KRAS(G12C) cells, but did not translate into a statistically significant difference of the ERCC1 protein level (Figure 5c).

Using EGFP-based reporter assays we measured HR, SSA, NHEJ and MMEJ activities. All KRAS mutant cells showed a significant DSB repair decrease in all the pathways compared to the KRAS(wt) cells (Figure 5d). We also investigated endogenous levels of key proteins involved in DSB repair and/or cisplatin responses. Western blot analysis did not reveal significant differences in the levels of 53BP1, BRCA1, KU70, MLH1 or RAD52 in the different clones, even though a trend to reduced KU70, MLH1 and RAD52 was noticed in the KRAS(G12C), for KU70 in the KRAS(G12D) and for MLH1 and RAD52 in the KRAS(G12V) clone. BACH1 levels were significantly lower in KRAS(G12C) and KRAS(G12D) compared with KRAS(wt) cells (*p* = 0.02) (Figure 5e). Altogether, NER and DSB repair-related features were unlikely to explain cisplatin resistance in KRAS(G12C) cells.

### Analysis of alternative DNA repair mechanisms in mutant KRAS expressing cells

Having so far failed to identify the mechanism responsible for KRAS(G12C)-mediated resistance to cisplatin, alternative DNA repair pathways with potential involvement in cisplatin adduct removal were investigated.

We examined the FA repair system with critical involvement in crosslink repair at stalled replication forks [20]. The mRNA expression analysis of FANCA, FANCC, FANCD2 and FANCF genes did not reveal any difference in the KRAS(G12C) clone possibly explaining cisplatin resistance (Figure 5f). Kinetics of FANCD2 foci assembly after cisplatin treatment were similar at least between KRAS(G12C) and KRAS(wt) or KRAS(G12D) clones (Figure 5g). FANCD2 activation was investigated by Western blot detection of ubiquitylation following cisplatin treatment. No significant differences were found, when determining the ratio between the activated (ubiquitylated) and inactive (not ubiquitylated) forms of the four clones (Figure 5h). PCNA ubiquitylation is triggered by replication stalling lesions such as DNA crosslinks, whereby mono-ubiquitylation leads to the polymerase switch between replicative and translesion synthesis polymerases, initiating another important repair process contributing to crosslink repair [20, 21]. PCNA mono-ubiquitylation as indicated by the appearance of a more slowly migrating band at 40 kDa was assessed after cisplatin treatment detecting mono-ubiquitylated protein in all clones (Figure 5i).

### Protective effect of BER against cisplatin-induced cytotoxicity in KRAS(G12C) cells

**Figure 6:**
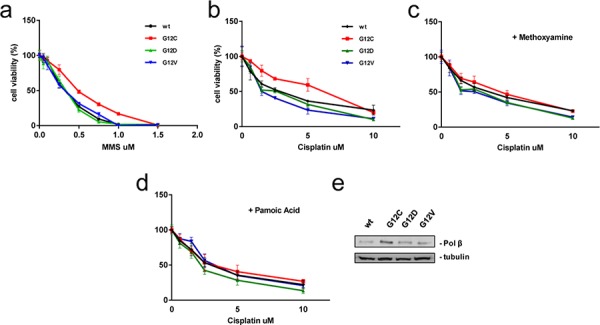
Protective effect of BER **a.** Response of cells to MMS treatment detected by MTS assay. The average of 3 independent experiments and SD are shown. Statistical analysis results are reported in Supplementary Table S1. **b–d.** Cells were continuously treated 72 h with increasing doses of cisplatin (b), cisplatin plus MA (c) and cisplatin plus PA (d) and vitality assessed by MTS assay. The average of 3 independent experiments and SD are shown. Statistical analysis results are reported in Supplementary Table S1. **e.** Representative Western blot image reporting the endogenous levels of Polβ. Tubulin was used as loading control and protein levels of KRAS(wt) clone were set to 1. Graphical presentation of Polβ levels is shown in Supplementary Figure S4c.

Finally, a potential role of BER in modulating cisplatin responsiveness was investigated. To this end, clones were treated with MMS that produces DNA methylation known to be processed by BER [22]. KRAS(G12C) clone resulted less sensitive to this compound when compared with the other clones suggesting a different activity of BER in clones (Figure 6a). We then treated cells with MA, a compound known to inhibit the initial incision step of BER [23]. Concomitant treatment of cells with MA at non toxic concentration, was able to almost completely restore the sensitivity of KRAS(G12C) clone to cisplatin (Figure 6b, 6c).

The expression of DNA polymerase beta (Polβ), the limiting component of BER activity, was evaluated by western blot and was found 2.7-fold overexpressed in the KRAS(G12C) compared to the KRAS(wt) clone (Figure 6e). Transfection with Polβ-specific siRNA successfully downregulated protein levels only after repeated Polβ siRNA transfection and only within 168 h (transfecting siRNA every 48 h) (Supplementary Figure S4a, S4b). This caused excessive cytotoxicity excluding the possibility to perform survival assays following siRNA-mediated Polβ knockdown. As an alternative approach, we applied PA, which was described as a potent Polβ inhibitor [24]. Pre-treatment of clones with PA at non-toxic concentration completely restored the sensitivity of KRAS(G12C) clone to the cisplatin treatment (Figure 6b, 6d) further confirming causal involvement of Polβ in altered cisplatin responsiveness.

Trying to elucidate the mechanism(s) by which Polβ was differently expressed in the clones, we analyzed the mRNA and protein stability. We could exclude a different Polβ protein stability among clones considering the experiments presented in Supplementary Figure S4a, S4b. The mRNA stability analysis of Polβ gene did not reveal as well any significant difference in the clones (Supplementary Figure S4d), whereas Polβ mRNA was more than 2-fold overexpressed in the KRAS(G12C) if compared to the KRAS(wt) (Supplementary Figure S4e), thus suggesting a transcriptional mechanism responsible for the increased expression of Polβ.

### Either BER and Polβ inhibitor treatments are able to rescue the phenotype of KRAS(G12C) cells

**Figure 7:**
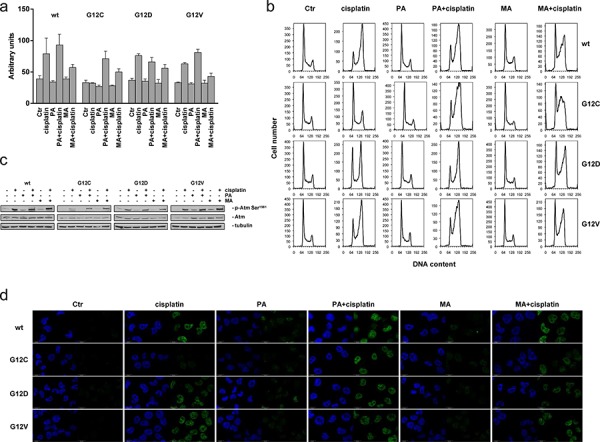
Rescue of the KRAS(G12C) cells phenotypes **a.** Caspase 3 and 7 activation: wild-type and mutant KRAS clones were treated with cisplatin, cisplatin plus MA and cisplatin plus PA. 24 h after treatment start caspase 3 and 7 activities were assessed by the Caspase-Glo 3/7 Assay. The average of 3 different biological replicates and SD are shown. Statistical analysis results are reported in Supplementary Table S1. **b.** Cell cycle phase distribution assessed 24 h after release from cisplatin. **c.** Representative Western blot analysis reporting the expression and phosphorylation of ATM on serine 1981 in cells 24 h after release from cisplatin. Tubulin was used as loading control. Graphical presentation of p-Atm Ser^1981^ levels from 2 experiments is shown in Supplementary Figure S4f **d.** Phosphorylation of H2AX histone (γH2AX, green) detected by immunofluorescence at 24 h after release from cisplatin, cisplatin plus MA and cisplatin plus PA. DAPI (blue) was used to counterstain the nuclei. Scale bar: 25 um.

In order to strengthen the role of BER and in particular of Polβ as responsible of all the findings described above, we applied either MA or PA in combination to cisplatin to rescue the phenotypes of KRAS(G12C) clone. Caspase 3/7, as previously reported, were not activated by cisplatin in the KRAS(G12C) clone. By applying either the PA or MA, although the latter was less effective, the caspase 3/7 were cleaved also in KRAS(G12C) clone (Figure 7a). Pre-treatment with PA or MA was also able to restore the ability of KRAS(G12C) cells to block the cell cycle and accumulate in G2/M upon cisplatin treatment as shown for other clones (Figure 7b). When we analyzed ATM activation, the less cisplatin sensitive KRAS(G12C) clone showed only a two-fold increase in the p-ATM compared to a 4–5-fold activation of the KRAS(wt), KRAS(G12D) and KRAS(G12V) clones 16–24 h after cisplatin treatment. By adding either MA or PA to cisplatin, the combinations were able to restore to activate ATM following cisplatin (Figure 7c, Supplementary Figure S4f). Similarly, the concomitant treatment with cisplatin and PA or MA restored the ability of KRAS(G12C) cells to activate H2AX (Figure 7d). Finally we set an experiment with a limited number of animals as a proof of principle for the activity of the combination cisplatin plus PA in xenograft model. Although the experiment was underpowered to have a statistical significant result, we observed an higher activity for the combination compared to cisplatin alone in the KRAS(G12C) clone injected mice. For the combination schedule we obtained a best T/C of 49% at day 14 compared to 70% of the cisplatin alone (data not shown).

### BER or Polβ inhibitor treatment is able to sensitize the KRAS(G12C) cells to cisplatin in other systems

To corroborate the potential role of KRAS(G12C) in modulating cisplatin responsiveness we extended our study to other systems.

The second independently generated NCI-H1299 KRAS(G12C) clone (cl.4), presented in Figure 1a, 1b and showing a lower response to cisplatin comparable to the KRAS(G12C) cl.2, was further investigated to confirm previous results. The KRAS(G12C) cl.4 was treated with MMS and, as reported for KRAS(G12C) cl.2, resulted less sensitive to this compound when compared with the wt clone (Supplementary Figure S5a). We then applied PA to restore the sensitivity to cisplatin. Pre-treatment with PA at non-toxic dose completely restored the sensitivity of KRAS(G12C) cl.4 to cisplatin (Supplementary Figure S5b) as previously reported for cl.2. We afterwards evaluated the expression of the Polβ and, as reported for the KRAS(G12C) cl.2, also the KRAS(G12C) cl.4 showed an overexpression of this gene both at mRNA and protein levels (Supplementary Figure S5c, S5d). By combining either the PA or MA to cisplatin, the ATM protein was activated also in KRAS(G12C) cl.4 (Supplementary Figure S5e). In the KRAS(G12C) cl.4, the γH2AX signals were almost undetectable 24 h after cisplatin treatment but the concomitant treatment with cisplatin and either PA or MA restored the ability of cells to activate H2AX (Supplementary Figure S5f). Cisplatin did not induce the caspase 3/7 cleavage in the KRAS(G12C) cl.4 as reported for cl.2 but this feature was rescued by applying either the Polβ inhibitor or MA although the latter was less effective (Supplementary Figure S5g).

To further strengthen our hypothesis on the role of KRAS(G12C), we generated and tested the influence of the different KRAS mutations in response to cisplatin treatment on mouse embryo fibroblasts NIH-3T3. Clones expressing comparable amount of KRAS(wt), KRAS(G12C), KRAS(G12D) and KRAS(G12V) were selected and treated with cisplatin. The clone expressing the KRAS(G12C) mutation showed a weaker response to cisplatin compared to KRAS(wt), KRAS(G12D) or KRAS(G12V) clones (Supplementary Figure S5h left). Concomitant treatment of cisplatin and PA at non toxic concentration, was able to completely restore the sensitivity of KRAS(G12C) clone to cisplatin (Supplementary Figure S5h right). As expected, the Polβ expression was higher at protein levels in the KRAS(G12C) clone when compared to the others (Supplementary Figure S5i).

Finally we compared two NSCLC cell lines harboring a different KRAS status, KRAS(wt) (NCI-H1299) or KRAS(G12C) (NCI-H358). The Polβ expression was higher, both at mRNA and protein levels, in the NCI-H358 cell line (Supplementary Figure S5j, S5k). The response to cisplatin was also weaker in the NCI-H358 cell line expressing the KRAS(G12C) mutant compared to KRAS(wt) expressing NCI-H1299 cells. When we applied PA we were able to induce, in the NCI-H358 cell line, a strong response to cisplatin comparable to the KRAS(wt) cell line NCI-H1299 (Supplementary Figure S5l) whereas PA treatment did not change the activity of cisplatin in the already sensitive cell line NCI-H1299.

## DISCUSSION

Platinum-based therapy remains so far the best treatment option for the majority of patients with NSCLC [25, 26]. Targeted therapy is available for a limited number of patients presenting specific mutations or translocations and these alterations are mutually exclusive with *KRAS* mutations [27–29]. This implies that patients with NSCLC presenting *KRAS* mutations are almost invariably treated in first-line with platinum-containing drugs. Given that *KRAS* mutations associate in several types of tumors with a more aggressive phenotype and/or resistance to treatment, [13, 30] it is mandatory to study the consequences of *KRAS* mutations on response to treatments. Mutations in the *KRAS* gene have been found mostly at codon 12 and 13 resulting in a pool of mutations differing in the replaced base and the substituted amino acid [31]. This spectrum of changes might be one of the reasons why mutated *KRAS* has so far not been an helpful marker to further classify patients in different cancer subgroups [32]. Here we provide evidence that cisplatin responsiveness indeed depends on the type of *KRAS* mutation. We found KRAS(G12C) mutant expressing cells, i.e. carrying the *KRAS* mutation most frequently found in NSCLC, to be the least responsive ones when compared with cells expressing other KRAS mutants or the KRAS(wt). These observations support our previous data indicating that cell lines expressing different *KRAS* mutations differently respond to drugs with different mechanisms of action [12]. The isogenic system we have used is based on a similar expression of exogenous KRAS (wt or mutated). The robustness of our systems is increased by the evidence that the introduction of exogenous KRAS (either wt or mutated) does not change the expression of endogenous KRAS (see Supplementary Figure S5m) which is similarly expressed in all the clones utilized avoiding the interference linked to the oncosuppressive role of KRAS(wt) as previously reported [33].

It is worth noting that the degree of resistance in our experimental settings is moderate, which, however, most likely reflects the clinical situation. Importantly, in this work, independently KRAS(G12C) expressing clones generated in different isogenic system (NCI-H1299 and NIH-3T3) showed a similar degree of cisplatin resistance when compared with KRAS(wt) clones, thus indicating that this finding is not restricted to a single cell line.

Analysis of the signaling pathways downstream of KRAS, such as involving MAPK and PI3K, [29] did not provide a clue for the peculiar response of KRAS(G12C) expressing cells to cisplatin. Similarly, another factor previously reported to be associated with resistance to cisplatin, namely increased detoxification through GST/GSH, [34, 35] did not play a significant role in this context. Reduced uptake or export of cisplatin can also underlie cisplatin resistance [36]. In our experimental model, cells expressing mutant compared to KRAS(wt) displayed a slight reduction in intracellular platinum but, as this was true for all three mutant clones, this aspect was unlikely to account for KRAS(G12C) clone-specific resistance to cisplatin.

Given a similar intracellular cisplatin level and comparable adduct formation on DNA at early time-points, downstream cellular activities had to account for the resistance of the KRAS(G12C) clone. Several pieces of evidence pointed to an impaired DNA damage response: i) analysis of the cell cycle perturbation induced by cisplatin treatment indicated only a weak G2/M phase block in KRAS(G12C) cells and this result was far different from all the other clones; ii) γH2AX foci formation following treatment was significantly reduced in the KRAS(G12C) clone compared to the others despite functionality of damage detection in these cells as demonstrated by experiments in which X-ray treatments induced γH2AX foci formation in a comparable way in the different clones and the KRAS(G12C) expressing clone also showed less DNA damage according to analyses of 53BP1-foci numbers and ATM activation; iii) finally, platinum bound to DNA disappeared completely within 24 h in KRAS(G12C) cells.

Altogether these data suggested that the KRAS(G12C) mutation stimulates a DNA repair mechanism promoting platinum removal from DNA before intra- and inter-strand crosslinks, avoiding cell growth arrest and/or death. Because levels of both DSB markers γH2AX and 53BP1 were reduced, this decisive DNA repair process might either be active before the formation of platinum cross-links which require cleavage and DSB formation at stalled replication forks or accelerate DSB repair itself [17, 20, 37].

To identify the molecular cause underlying the reduced drug response of KRAS(G12C) cells, we investigated different DNA repair mechanisms known to play a role in the response to cisplatin. Clones expressing mutated compared to KRAS(wt) showed reduced capacity of the more error-free DSB repair activities HR and canonical NHEJ as well as of the error-prone pathways SSA and MMEJ. As it applied to all three KRAS mutant clones, this feature was unlikely to explain cisplatin resistance in KRAS(G12C) expressing cells. These findings were corroborated by the analysis of the expression of proteins of interest involved in the different DSB repair pathways.

Other DNA repair mechanisms that were demonstrated to play a role in cisplatin damage repair, and thus were candidate systems for the removal of adducts from DNA were NER and the FA pathway [17, 20, 35, 38]. However, our expression and functional data addressing these pathways excluded their involvement in the cisplatin resistance mechanism of KRAS(G12C) expressing clones. Of note, the KRAS(G12C) clone was, among the different clones tested, the most susceptible to UV light suggesting, if any, a less active NER system. Even mismatch repair has been implicated in the cisplatin response, namely in mediating cytotoxicity [39]. Notably, the mismatch repair protein MLH1 had previously been found to become downregulated by promoter methylation in a significant fraction of NSCLC [40] but comparable levels of MLH1 protein were detected in different KRAS expressing clones in our work.

Here, we provide several pieces of evidence supporting the idea that short-patch BER involving Polβ could be involved in the different behavior of KRAS(G12C) mutant expressing cells once treated with cisplatin.

Higher Polβ levels detected in the KRAS(G12C) compared with the other cells may stimulate BER activity resulting in a fast platinum adducts removal from the DNA. This feature would prevent intra- and inter-strand crosslink allowing these cells to growth and survive. Restoration of cisplatin sensitivity in the resistant KRAS(G12C) clone after BER/Polβ specific inhibitors treatment supported the idea that BER and in particular Polβ activity may account for cisplatin resistance in these cells. Interestingly, co-treatment with BER or Polβ specific inhibitors was also able to rescue the altered (compared to the other KRAS mutants and wt cells) phenotype of KRAS(G12C) cells, restoring apoptosis, cell cycle perturbation, γH2AX foci formation and ATM activation.

Elevated levels of Polβ have been associated with resistance to cisplatin treatment in colon cancer [41] and high levels of Polβ have been found in many cancers including breast, colon, ovarian and prostate cancers [42–45]. Metabolic changes in tumors lead to oxidative stress [46] and thus oxidatively damaged DNA requiring higher BER activities and elevated levels of the limiting component Polβ for survival, which concomitantly result in resistance to therapy.

We established a link between KRAS(G12C) and Polβ, showing that cells expressing this specific mutant have an increased expression of Polβ likely due to an increased transcription of the gene. Polβ gene transcription is regulated by several transfactors (including ATF/CREB family members) and mutations in the binding sites present in its promoter affect its transcription [47, 48]. Very recently a direct link between CREB and KRAS has been postulated [49] thus making possible the hypothesis that a different interaction between different KRAS mutants and CREB could be responsible for the increased transcription observed in KRAS(G12C) mutants cells. This hypothesis will be actively pursued in the future.

At present, there is little information regarding Polβ expression in *KRAS* mutated lung cancer, so that this biomarker will have to be assessed in this type of cancer in the future. Importantly, when testing sensitivity to different inhibitors of PARP, which has been proposed to be involved in BER and to target HR-defective cells, [50] we observed increased sensitivity rather than resistance of mutant KRAS including KRAS(G12C) expressing cells (data not shown). Thus, PARP inhibition may overcome excessive BER in KRAS(G12C) expressing cells and at the same time target HR deficiency in all mutant KRAS cells. Therefore, the possibility remains that PARP inhibition could represent an additional therapeutic option for NSCLC in combination treatment approaches including combined cisplatin and PARP inhibitor regimens [51].

In conclusion our data demonstrate and confirm that different *KRAS* mutations have a different impact on cisplatin sensitivity. This information will have to be taken into account when designing new clinical studies aiming at the evaluation of the role of *KRAS* as prognostic and predictive marker in NSCLC. Classification of tumors solely by the presence of a mutation in *KRAS*, without defining the specific mutation, might not be enough to identify patients with a different response to therapy in cancers harboring a *KRAS* mutation. Our preclinical models represent an important tool to test new therapeutic strategies which could represent the starting point for the design of new trials in the clinical setting.

## MATERIALS AND METHODS

### Cell cultures and drug treatments

The NIH3T3, NCI-H1299 and NCI-H358 were purchased by ATCC. The NIH-3T3 was grown in DMEM, the NCI-H1299 and NCI-H358 were grown in RPMI-1640 medium. Clones were obtained by transfecting the NCI-H1299 and NIH-3T3 cell line with the expression plasmids encoding for the different mutations (G12C, G12D and G12V) and the wt KRAS as a control. All clones were grown in medium including 500 ug/ml of G418 (Gibco). Cells are routinely tested for mycoplasma contamination by PCR and authenticated with the PowerPlex 16 HS System (Promega) every 6 months by comparing the STR profiles to which deposited in ATCC and/or DSMZ databases. Cisplatin, melphalan, PA, MA, MMS (Sigma Aldrich), isoquinolinediol (Calbiochem) and NU1025 (Enzo Life Sciences) were dissolved in medium just before use. Treatments, unless otherwise specified, were performed at 5 uM for 2 h or 1.5 uM for 24 h for cisplatin, 200 uM for MA and 100 uM for PA. The MTS assays (Promega) were performed as described in [12]. Clonogenic assays were performed as described in [52]. Survival curves, unless otherwise specified, were plotted as percentages of untreated controls, consisted of at least 6 replicates for each time point and represented the average mean and SD of at least 3 independent experiments.

### Western blotting analysis

Proteins were extracted and visualized as reported in [52, 53]. Immunoblotting was carried with the antibodies reported in the Supplementary Information. Protein bands of interest were quantified using Image Lab 4.1 (Bio-Rad Laboratories) or ImageJ 1.48 (NIH) softwares and corrected with the values obtained for the loading control each.

### KRAS activation assay

The active form of KRAS was measured with KRAS Activation assay Kit (Cell Biolabs) according to the manufacturer's instructions.

### Immunofluorescence microscopy and foci count

Immunofluorescence microscopy were performed as described in [53] and [54]. Primary antibodies used are reported in the Supplementary Information. Immunolabeled foci were scored by automated quantification and mean numbers of foci per cellular nucleus in the total cell population calculated from 4 slides analyzing 200 nuclei in total.

### Real time PCR

Total RNA was reverse transcribed with High-Capacity cDNA Kit (Life Technologies) and amplified by 7900 HT Sequence Detection System (Life Technologies). Actin was used as internal control. Primers and TaqMan probes were purchased for all genes as ready-to-use solutions (Life Technologies). Two samples which showed at least 2-fold differences were considered differently expressed.

### *In vivo* activity

Procedures involving animals were described in [52] and their care are reported in the Supplementary Information section.

For these specific experiments female athymic NCr-*nu/nu* mice, seven weeks old, obtained from Harlan Laboratories were inoculated s.c. with 200 ul of cell suspension containing 10^7^ cells. When the average of the tumor weights reached about 200 mg (excluding animals with tumors <100 mg or >400 mg in weight), mice were randomized. Cisplatin was given intravenously at the dose of 5 mg/kg, every 7d for three times (q7dx3). Each group comprised 8 mice. The investigator who performed the *in vivo* studies was not informed about the *in vitro* results regarding cisplatin citotoxicity. A T/C < 42% is considered the minimum level for activity [55].

### Caspases 3 and 7 activity assay

Twenty-four hours after cells plating, drug treatment was performed and 24 h or 48 h later caspase activity was assessed using the Caspase-Glo 3/7 Assay (Promega) according to the manufacturer's instructions.

### Monoparametric staining of DNA content

Sample preparation and monoparametric DNA histograms analysis were performed as described in [56].

### Measurement of platinum content

Equal numbers of cells were resuspended in 200 ul of PBS and 400 ul of HNO_3_:HCl (1:3). After 12 h at RT, 600 ul of water were added, vortexed and centrifuged at 16,000 × g for 10 min at 4°C. The supernatant was injected into Analyst 600 (Perkin Elmer). A calibration curve with platinum standard (Sigma Aldrich) was generated.

Platinum bound to DNA was determined with DRC-ICP-MS using an ELAN DRC (Perkin Elmer) equipped with cyclonic spray chamber and a Meinhard type concentric nebulizer. The uncertainty of measurements was evaluated as suggested by international bodies (ISO and EURACHEM/CITAC).

### Determination of DSB repair frequencies

Fluorescence-based DSB repair measurements were performed for different DSB repair pathways following targeted cleavage by I-Sce I-meganuclease as described in [57, 58].

### Statistical analyses

The Statistical analyses were performed using GraphpadPrism version 5.01. Specific tests used to analyze specific experiments are indicated in the Supplementary Table S1. Differences between groups were considered statistically significant when the *p*-values were ≤ 0.05.

## SUPPLEMENTARY INFORMATION FIGURES AND TABLES



## Abbreviations

DRC-ICP-MS: Dynamic Reaction Cell Inductively Coupled Plasma Mass Spectrometry
NSCLC: non-small-cell lung cancer
MAPK: mitogen-activated protein kinase
PI3K: phosphoinositide 3-kinase
GST: glutathione S-transferase
GSH: glutathione
CTR-1: Copper transporter-1
DSB: double strand break
NER: nucleotide excision repair
HR: homologous recombination
SSA: single-strand annealing
NHEJ: non-homologous end-joining
MMEJ: micro-homology mediated NHEJ
FA: Fanconi anemia
BER: base excision repair
wt: wild-type
EGFR: epidermal growth factor receptor
ALK: Anaplastic lymphoma kinase
KRAS: V-Ki-ras2 Kirsten rat sarcoma viral oncogene homolog
ATM: protein kinase ataxia-telangiectasia mutated
MMS: Methyl methanesulfonate
cl.: clone
MA: methoxyamine
PA: Pamoic acid
