# Supplementary material for: Base excision repair-mediated resistance to cisplatin in KRAS(G12C) mutant NSCLC cells
Source: Oncotarget. 2015 Sep 2;6(30):30072–87. doi: 10.18632/oncotarget.5019 (PMC4745782; doi:10.18632/oncotarget.5019)
Supplement: Supplementary file 1 [file oncotarget-06-30072-s001.pdf]

SUPPLEMENTARY INFORMATION

Antibodies used in the study for western blotting analysis

anti-Akt #9272, anti-Akt(Ser473) #9271, anti-Akt(Thr308) #9275, anti-p70S6K(Thr389) #9206, anti-p70S6K #9202, anti-S6(Ser235/236) ribosomal protein #2211, anti-S6 ribosomal protein 2217#, anti-4E-BP1(Thr37/46) #2855, anti-4E-BP1 #9644, anti-PRAS40(Thr^{246}) #2997, anti-PRAS40 #2691, anti-Mek1/2(Ser$^{217/221}$) #9154, anti-Mek1/2 #9122 provided by Cell Signalling Technology. Anti-Atm(Ser^{1981}) #2152–1 was obtained from Epitomics. Anti-Atm #sc23921, Anti-Actin #sc1615, anti-Erk #sc94, anti-Erk(Tyr^{204}) #sc7383, anti-KRAS #sc30, anti-FancD2 #sc20022, and anti-Lamin B #sc6217 were obtained from Santa Cruz Biotechnology. Anti-53BP1 #NB100–304 (Novus Biologicals), anti-BACH1 #B1310 (Sigma), anti-BRCA1 #Ab-1 (Calbiochem), anti-ERCC1 #8F1 and anti-MLH1 #G168–15 (Becton Dickinson), anti-DNA polymerase beta #ab26343, anti-KU70 #S5C11, anti-PCNA #PC10 provided by Abcam, anti-RAD52 #Ab-1 (Neomarkers).

Antibodies used in the study for Immunofluorescence microscopy and foci count

anti-53BP1 #NB100–304 (Novus Biologicals), anti-FANCD2 #ab2187 (Abcam), anti-RAD51 #H-92 (Santa Cruz Biotechnology), anti-BRCA1 #Ab-1 (Calbiochem), anti-γH2AX #JBW301 (Merck Millipore,).

In vivo activity

Procedures involving animals and their care were conducted in conformity with the following laws, regulations, and policies governing the care and use of laboratory animals: Italian Governing Law (D.lgs 26/2014; Authorisation n.19/2008-A issued March 6, 2008 by Ministry of Health), Mario Negri Institutional Regulations and Policies providing internal authorisation for persons conducting animal experiments (Quality Management System Certificate – UNI EN ISO 9001:2008 – Reg. N° 8576-A), the NIH Guide for the Care and Use of Laboratory Animals (2011 edition), the EU directives and guidelines (EEC Council Directive 2010/63/UE) and in line with Guidelines for the welfare and use of animals in cancer research. Animal experiments has been reviewed and approved by the IRFMN Animal Care and Use Committee (IACUC) that includes members "ad hoc" for ethical issues. Animals were housed in the Institute's Animal Care Facilities, which meet international standards; they are regularly checked by a certified veterinarian who is responsible for health monitoring, animal welfare supervision, experimental protocols and procedures revision.

REFERENCE

1. Workman P, Aboagye EO, Balkwill F, Balmain A, Bruder G, Chaplin DJ, et al. Guidelines for the welfare and use of animals in cancer research. Br J Cancer. 2010; 102:1555–1577.

SUPPLEMENTARY FIGURES AND TABLES

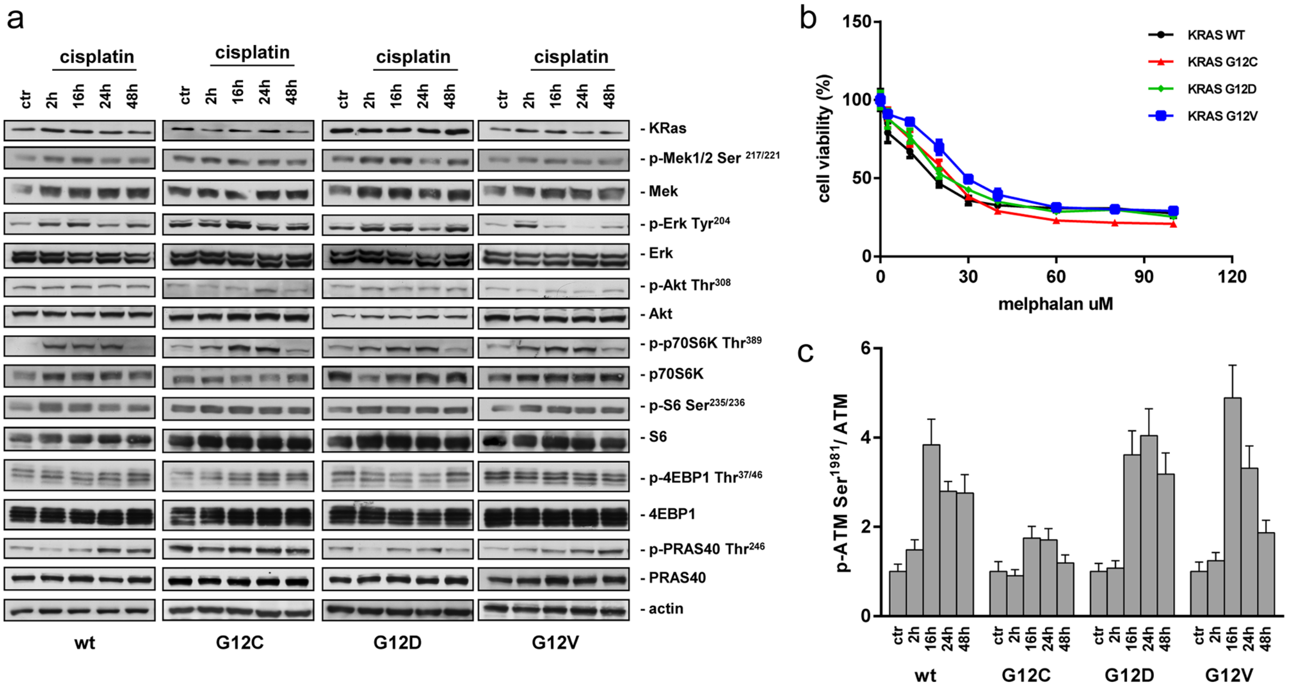

Supplementary Figure S1: a. MAPK and PI3K signalling. Representative Western blot analysis reporting the expression of different proteins belonging to the MAPK and PI3K pathways in the KRAS expressing clones treated with cisplatin estimated at the indicated time points. Actin was used as loading control. Two independent experiments have been performed. **b.** Growth inhibition assay: response of cells to melphalan treatment detected by MTS assay. The average of 3 independent experiments and SD are shown. Statistical analysis results are reported in Supplementary Table S1. **c.** Graphical presentation of ATM phosphorylation. Band intensities of p-ATM were quantified and individually normalized with unmodified ATM band intensities. Protein levels of KRAS(wt) expressing cells were set to 1 each. Quantification from 2 independent Western blots (means, SD) are shown. Statistical analysis results are reported in Supplementary Table S1.

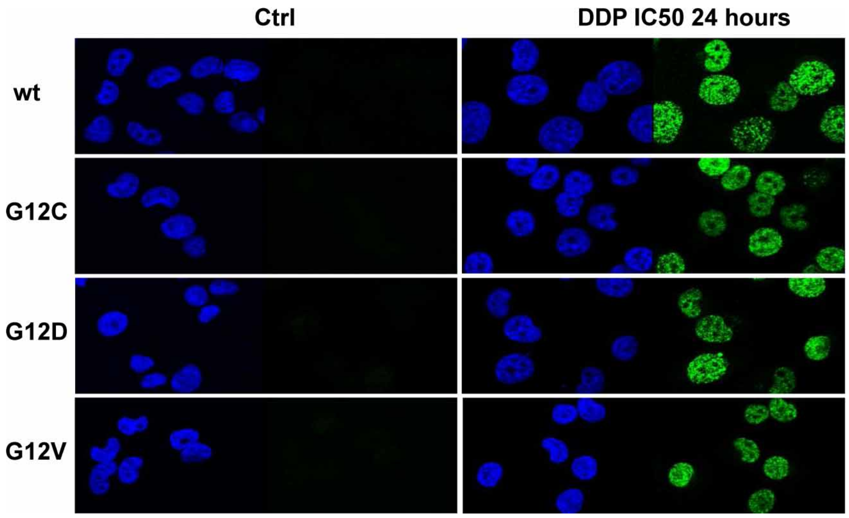

Supplementary Figure S2: Phosphorylation of H2AX (green) detected by immunofluorescence microscopy 24 h after cisplatin treatment for 2 h at IC50 for each clone. DAPI (blue) was used to counterstain the nuclei. Scale bar: 25 um.

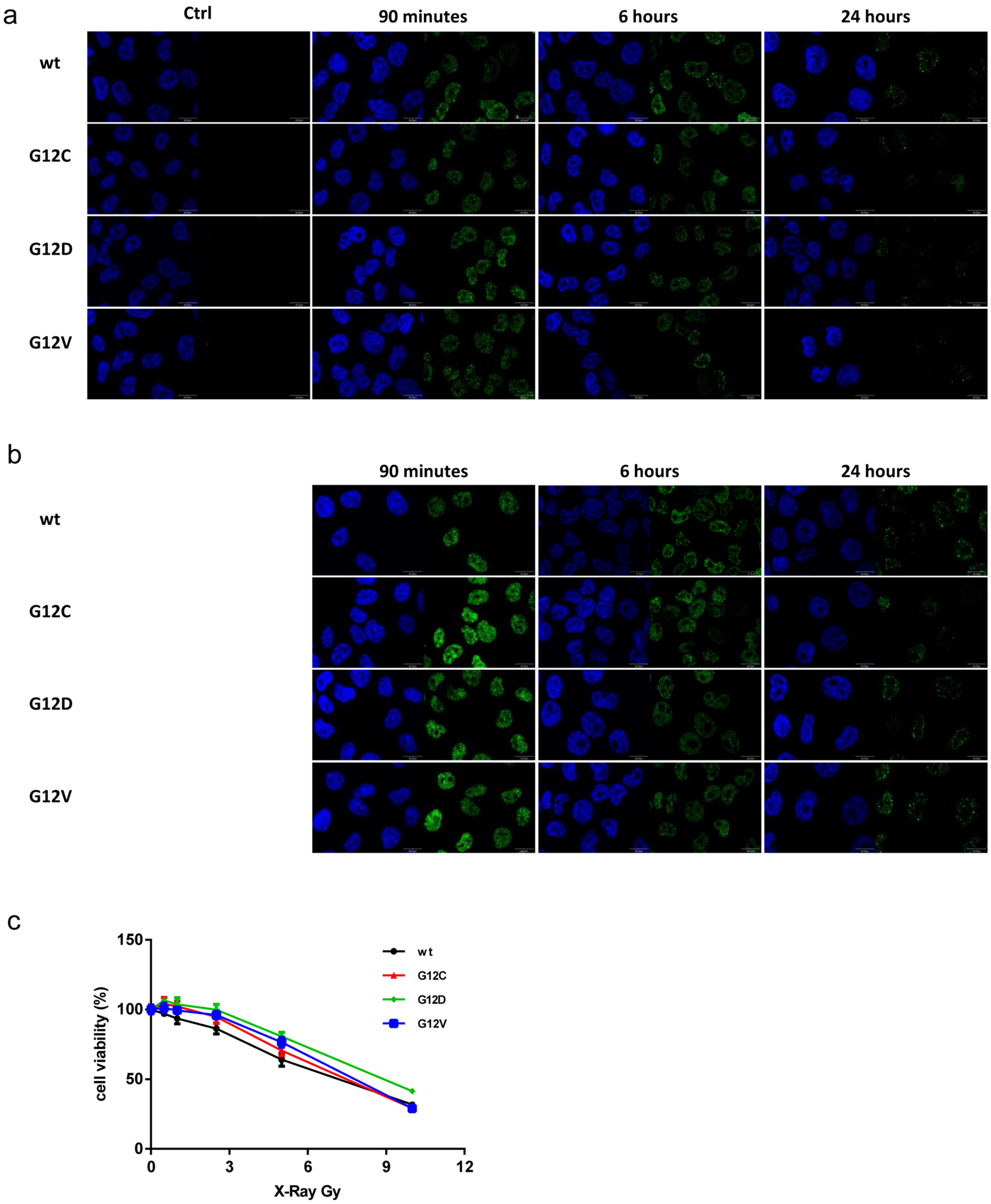

Supplementary Figure S3: Phosphorylation of H2AX after treatment with ionizing radiation (IR). a–b. Phosphorylation of histone H2AX (green) detected by immunofluorescence microscopy at the indicated time points after X-ray treatment with 5 (a) and 7.5 (b) Gy. DAPI (blue) was used to counterstain the nuclei. Scale bar: 25 um. **c.** Growth inhibition assay: response of cells to X-ray treatment detected by MTS assay. The average of 3 independent experiments and SD are shown. Statistical analysis results are reported in Supplementary Table S1.

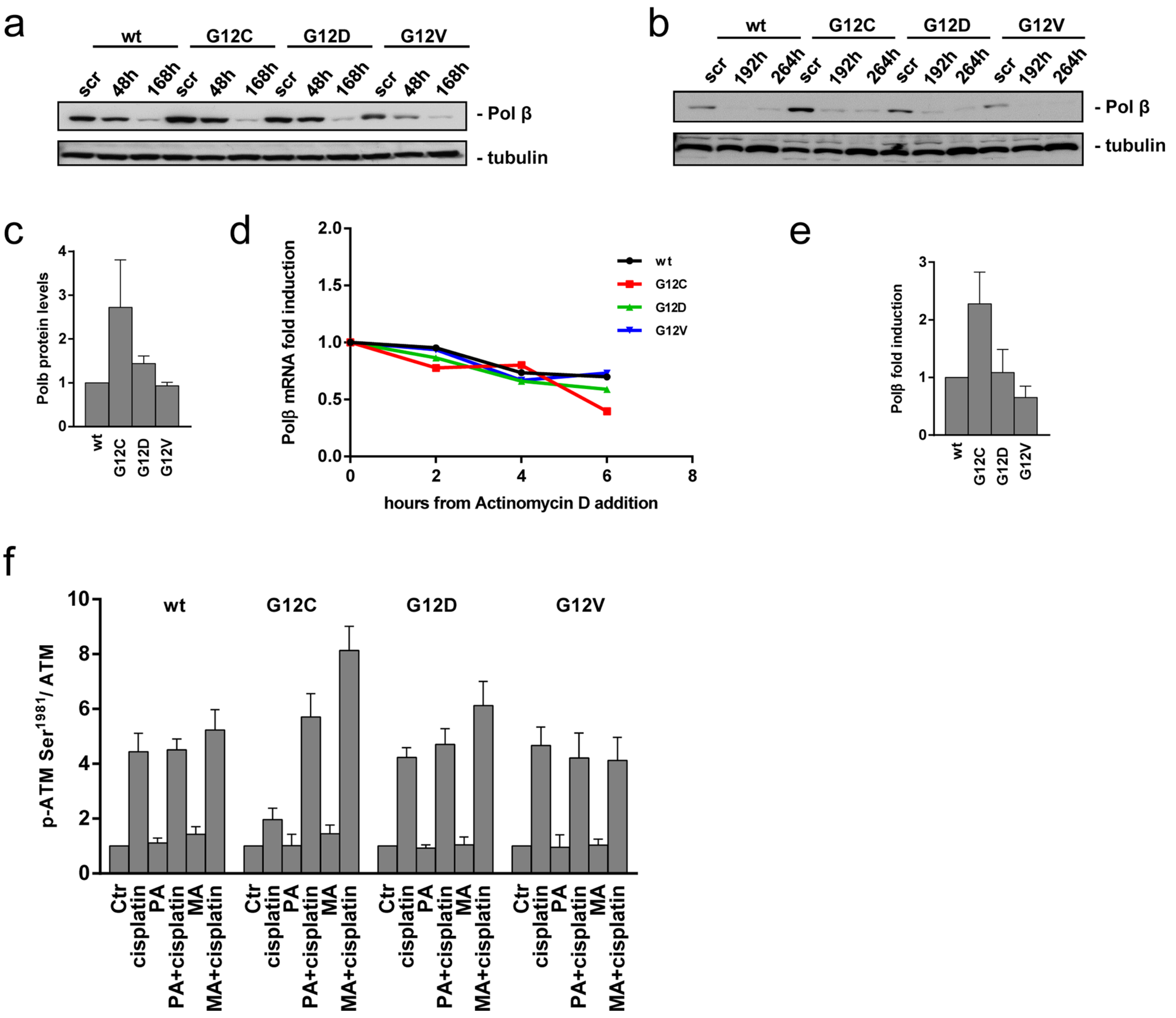

Supplementary Figure S4: **a.** Representative Western blot analysis showing Polβ expression 48 h and 168 h following negative control or Polβ siRNA transfection every 48 h. Tubulin was used as loading control. Two independent experiments have been performed. **b.** Representative Western blot analysis showing Polβ expression 192 h and 264 h following negative control or Polβ siRNA transfection every 48 h. Tubulin was used as loading control. Two independent experiments have been performed. **c.** Graphical presentation of Polβ. Band intensities of Polβ were quantified and individually normalized with tubulin band intensities. Protein levels of KRAS(wt) expressing cells were set to 1. Quantification from 5 independent Western blots (means, SD) are shown. Statistical analysis results are reported in Supplementary Table S1. **d.** Polβ mRNA half-life determined by real time PCR at different time points after addition of actinomycin D 10 ug/ml. **e.** Relative mRNA expression levels of Polβ measured by real time PCR in KRAS expressing clones at basal level. Values for the KRAS(wt) clone were set to 1. The average of 2 independent experiments and SD are shown.

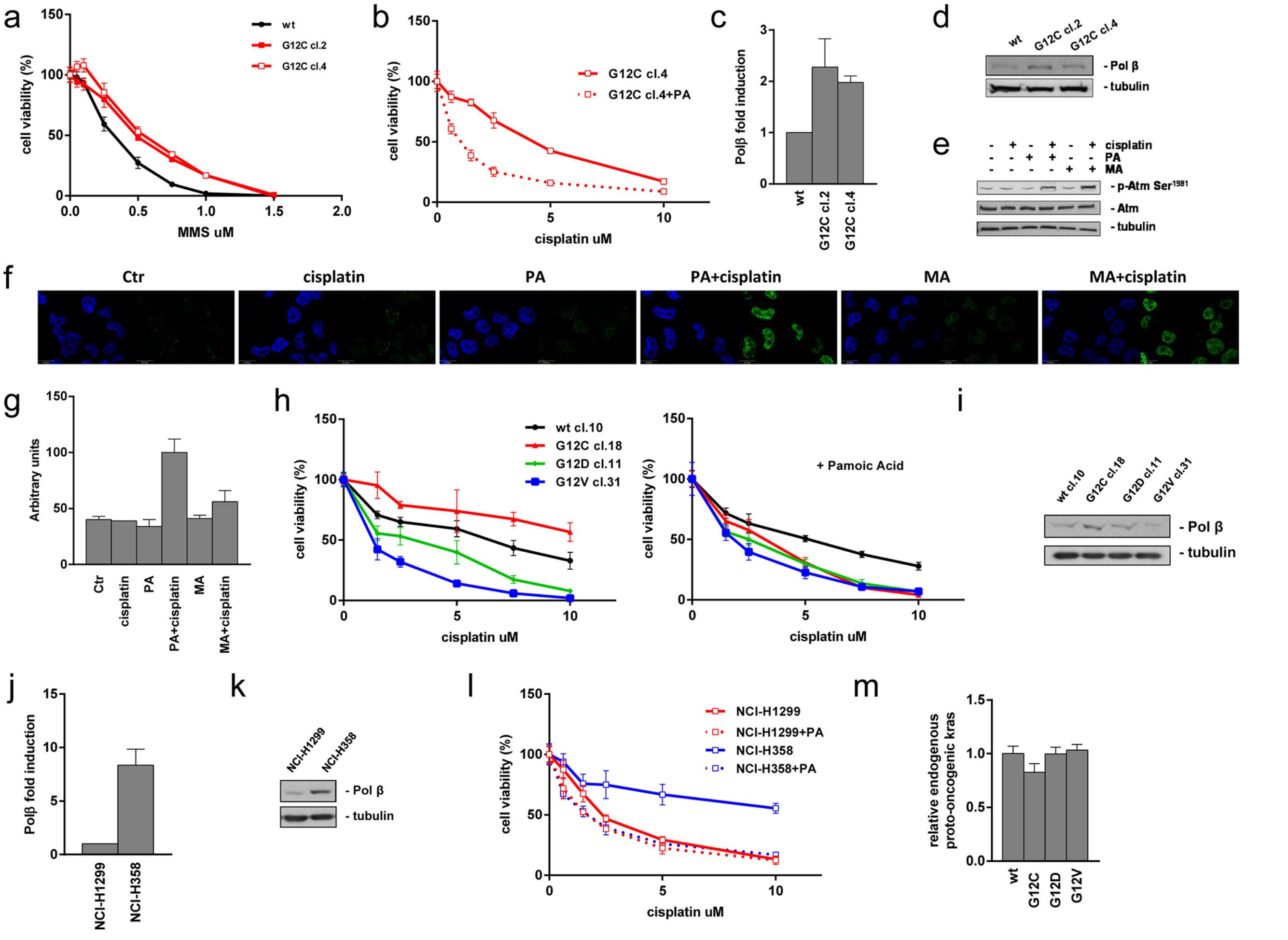

Supplementary Figure S5: a. Growth inhibition assay: response of cells to MMS treatment detected by MTS assay. The average of 3 independent experiments and SD are shown. Statistical analysis results are reported in Supplementary Table S1. **b.** Growth inhibition assay: response of cells to cisplatin or cisplatin plus PA treatment detected by MTS assay. The average of 3 independent experiments and SD are shown. **c.** Relative mRNA expression levels of Polβ measured by real time PCR in KRAS clones at basal level. Values for the KRAS(wt) clone were set to 1. The average of 3 different biological replicates and SD are shown. **d.** Representative Western blot image reporting the endogenous levels of Polβ. Tubulin was used as loading control. **e.** Representative Western blot analysis reporting the expression of and phosphorylation of ATM on serine 1981 in cells at 24 h after release from cisplatin. Tubulin was used as loading control. **f.** Phosphorylation of H2AX histone (γH2AX, green) in KRAS(G12C) cl.4 detected by immunofluorescence at 24 h after release from cisplatin, cisplatin plus MA and cisplatin plus PA. DAPI (blue) was used to counterstain the nuclei. Scale bar: 25 um. **g.** Caspase 3 and 7 activation: KRAS(G12C) cl.4 was treated with cisplatin, cisplatin plus MA and cisplatin plus PA. 24 h after recovery caspase 3 and 7 activities were assessed by the Caspase-Glo 3/7 Assay. The average of 3 different biological replicates and SD are shown. Statistical analysis results are reported in Supplementary Table S1. **h.** Growth inhibition assay: response of cells to cisplatin or cisplatin plus PA treatment detected by MTS assay. The average of 3 independent experiments and SD are shown Statistical analysis results are reported in Supplementary Table S1. **i.** Representative Western blot image reporting the endogenous levels of Polβ in NIH-3T3 fibroblasts expressing different KRAS mutations. Tubulin was used as loading control. **j.** Relative mRNA expression levels of Polβ measured by real time PCR in NSCLC cell lines at basal level. Values for the NCI-H1299 were set to 1. The average of 2 different biological replicates and SD are shown. **k.** Representative Western blot image reporting the endogenous levels of Polβ in NSCLC cell lines Tubulin was used as loading control. **l.** Growth inhibition assay: response of cells to cisplatin or cisplatin plus PA treatment detected by MTS assay. The average of 3 independent experiments and SD are shown. **m.** Relative mRNA expression levels of endogenous KRAS measured by real time PCR in NCI-H1299 derived clones at basal level. Values for the wt were set to 1. The average of 2 different biological replicates and SD are shown.

Supplementary Table S1: Statistical differences of data presented

p*-value < 0.05, ***p*-value < 0.01, ****p*-value < 0.001,***p*-value < 0.0001

Figure 1b: Growth inhibition assay: response of cells to cisplatin treatment.

	Cisplatin uM								
	1	2,5	5	7,5	10	15	20	25	30
wt cl4 vs. wt cl11	ns	ns	ns	ns	ns	ns	ns	ns	ns
wt cl4 vs. G12C cl2	ns	ns	ns	*	ns	****	***	***	****
wt cl4 vs. G12C cl4	ns	ns	ns	*	ns	**	*	ns	***
wt cl4 vs. G12D cl2	ns	ns	ns	ns	ns	ns	ns	ns	ns
wt cl4 vs. G12D cl3	ns	ns	ns	ns	ns	ns	ns	ns	ns
wt cl4 vs. G12V cl9	ns	ns	ns	**	***	ns	****	**	ns
wt cl4 vs. G12V cl22	ns	ns	ns	*	****	*	***	***	*
wt cl11 vs. G12C CL2	ns	ns	*	ns	**	**	***	****	****
wt cl11 vs. G12C CL4	ns	ns	*	ns	**	ns	*	**	**
wt cl11 vs. G12D CL2	ns	ns	ns	ns	ns	ns	ns	ns	ns
wt cl11 vs. G12D CL3	ns	ns	ns	ns	ns	ns	ns	ns	ns
wt cl11 vs. G12V cl9	ns	ns	ns	**	*	**	****	*	*
wt cl11 vs. G12V cl22	ns	ns	ns	**	**	****	***	**	**
G12C CL2 vs. G12C CL4	ns	ns	ns	ns	ns	ns	ns	ns	ns
G12C CL2 vs. G12D CL2	ns	ns	*	**	*	**	****	****	****
G12C CL2 vs. G12D CL3	ns	ns	ns	**	ns	***	***	****	****
G12C CL2 vs. G12V CL9	ns	ns	***	****	****	****	****	****	****
G12C CL2 vs. G12V CL22	ns	ns	***	****	****	****	****	****	****
G12C CL4 vs. G12D CL2	ns	ns	*	**	*	ns	**	*	****
G12C CL4 vs. G12D CL3	ns	ns	ns	**	ns	*	*	**	****
G12C CL4 vs. G12V CL9	ns	ns	***	****	****	****	****	****	****
G12C CL4 vs. G12V CL22	ns	ns	***	****	****	****	****	****	****
G12D CL2 vs. G12D CL3	ns	ns	ns	ns	ns	ns	ns	ns	ns
G12D CL2 vs. G12V CL9	ns	ns	ns	*	**	**	***	**	ns

	Cisplatin uM								
	1	2,5	5	7,5	10	15	20	25	30
G12D CL2 vs. G12V CL22	ns	ns	ns	ns	***	****	**	***	ns
G12D CL3 vs. G12V CL9	ns	ns	ns	*	****	*	****	ns	ns
G12D CL3 vs. G12V CL22	ns	ns	ns	ns	****	***	***	*	ns
G12V CL9 vs. G12V CL22	ns	ns	ns	ns	ns	ns	ns	ns	ns

Statistical analysis was performed using two-way ANOVA test and Bonferroni post-test for multiple comparisons.

Figure 1c: Colony formation assay: response of cells to different doses of cisplatin

	Cisplatin uM				
	1	2,5	5	10	20
wt vs. G12C	ns	***	*	*	ns
wt vs. G12D	ns	ns	ns	ns	ns
wt vs. G12V	ns	ns	ns	ns	ns
G12C vs. G12D	****	****	**	****	*
G12C vs. G12V	ns	**	**	*	ns
G12D vs. G12V	***	*	ns	ns	ns

Statistical analysis was performed using two-way ANOVA test and Bonferroni post-test for multiple comparisons.

Figure 1d: Caspase 3 and 7 activation: wt and mutant KRAS clones were treated with cisplatin.

	Time after treatment start		
	0	24 h	48 h
wt vs. G12C	ns	****	****
wt vs. G12D	ns	**	**
wt vs. G12V	ns	*	ns
G12C vs. G12D	ns	ns	****
G12C vs. G12V	ns	**	****
G12D vs. G12V	ns	ns	ns

Statistical analysis was performed using two-way ANOVA test and Bonferroni post-test for multiple comparisons.

Figure 2b–c: Tumor growth inhibition activity on KRAS(wt) or KRAS(G12C) expressing clones injected mice.

	Days after tumor implant						
	27	28	32	35	39	42	45
wt ctr vs. wt cisplatin	ns	ns	***	ns	****	****	****
G12C ctr vs. G12C cisplatin	ns	ns	ns	ns	ns	ns	**

Statistical analysis was performed using two-way ANOVA test and Bonferroni post-test for multiple comparisons.

Figure 2d: Assessment of intracellular platinum concentration at the indicated time points after cisplatin treatment.

	Time after treatment start				
	**2 h**	**6 h**	**16 h**	**24 h**	**48 h**
wt vs. G12C	*	ns	***	ns	ns
wt vs. G12D	****	**	***	**	ns
wt vs. G12V	ns	ns	***	ns	ns
G12C vs. G12D	ns	ns	ns	ns	ns
G12C vs. G12V	ns	ns	ns	ns	ns
G12D vs. G12V	*	ns	ns	ns	ns

Statistical analysis was performed using two-way ANOVA test and Bonferroni post-test for multiple comparisons.

Figure 2f: Assessment of platinum adducts bound to DNA at the indicated time points after cisplatin treatment.

	Time after treatment start		
	**2 h**	**6 h**	**24 h**
wt vs. G12C	ns	ns	**
wt vs. G12D	ns	*	ns
wt vs. G12V	ns	**	**
G12C vs. G12D	ns	ns	*
G12C vs. G12V	ns	ns	***
G12D vs. G12V	ns	ns	ns

Statistical analysis was performed using multiple *t*-test.

Figure 3e: γH2AX immunolabeled foci from 2–4 slides and two independent experiments.

	Time after release from treatment		
	**6 h**	**16 h**	**24 h**
wt vs. G12C	***	****	****
wt vs. G12D	ns	****	ns
wt vs. G12V	ns	****	ns
G12C vs. G12D	*	**	****
G12C vs. G12V	**	**	****
G12D vs. G12V	ns	ns	ns

Statistical analysis was performed using two-way ANOVA test and Bonferroni post-test for multiple comparisons.

Figure 4b: Focal accumulation of DSB repair proteins after cisplatin treatment: 53BP1.

	Time after release from treatment		
	6 h	16 h	24 h
wt vs. G12C	ns	****	****
wt vs. G12D	ns	**	ns
wt vs. G12V	ns	****	****
G12C vs. G12D	ns	****	****
G12C vs. G12V	ns	****	****
G12D vs. G12V	ns	*	****

Statistical analysis was performed using two-way ANOVA test and Bonferroni post-test for multiple comparisons.

Figure 4d: Focal accumulation of DSB repair proteins after cisplatin treatment: BRCA1.

	Time after release from treatment		
	6 h	16 h	24 h
wt vs. G12C	ns	ns	****
wt vs. G12D	ns	ns	ns
wt vs. G12V	ns	**	ns
G12C vs. G12D	ns	ns	****
G12C vs. G12V	ns	****	**
G12D vs. G12V	ns	**	ns

Statistical analysis was performed using two-way ANOVA test and Bonferroni post-test for multiple comparisons.

Figure 4e: Focal accumulation of DSB repair proteins after cisplatin treatment: RAD51.

	Time after release from treatment		
	6 h	16 h	24 h
wt vs. G12C	ns	ns	ns
wt vs. G12D	ns	ns	ns
wt vs. G12V	ns	***	ns
G12C vs. G12D	ns	ns	ns
G12C vs. G12V	ns	*	ns
G12D vs. G12V	ns	***	ns

Statistical analysis was performed using two-way ANOVA test and Bonferroni post-test for multiple comparisons.

Figure 5a: Growth inhibition assay: response of cells to treatment with UV light.

	UV light J/m2					
	2	5	10	15	20	25
wt vs. G12C	*	*	*	**	**	**
wt vs. G12D	**	ns	ns	ns	ns	ns
wt vs. G12V	ns	ns	ns	ns	ns	ns
G12C vs. G12D	ns	ns	ns	ns	ns	ns
G12C vs. G12V	**	ns	ns	ns	ns	ns
G12D vs. G12V	***	ns	ns	ns	ns	ns

Statistical analysis was performed using two-way ANOVA test and Bonferroni post-test for multiple comparisons.

Figure 5d: DSB repair frequencies.

	HR	SSA	NHEJ	MMEJ
wt vs. G12C	**	****	****	****
wt vs. G12D	*	****	****	****
wt vs. G12V	**	****	****	****
G12C vs. G12D	ns	ns	ns	**
G12C vs. G12V	ns	ns	ns	ns
G12D vs. G12V	ns	ns	ns	ns

Statistical analysis was performed using one-way ANOVA test and Bonferroni post-test for multiple comparisons.

Figure 5e: Expression of DSB repair proteins.

	BACH1
wt vs. G12C	****
wt vs. G12V	ns
wt vs. G12D	**
G12C vs. G12V	**
G12C vs. G12D	ns
G12V vs. G12D	*

Statistical analysis was performed using one-way ANOVA test and Bonferroni post-test for multiple comparisons.

Figure 5g: FANCD2 immunolabeled foci from 2 slides from independent experiments.

	Time after release from treatment		
	6 h	16 h	24 h
wt vs. G12C	ns	ns	ns
wt vs. G12D	ns	ns	*
wt vs. G12V	**	****	**
G12C vs. G12D	ns	ns	ns
G12C vs. G12V	***	****	ns
G12D vs. G12V	***	****	ns

Statistical analysis was performed using two-way ANOVA test and Bonferroni post-test for multiple comparisons

Figure 6a: Growth inhibition assay: response of cells to MMS treatment detected by MTS assay.

	MMS uM						
	0.05	0.1	0.25	0.5	0.75	1	1.5
wt vs. G12C	ns	ns	****	****	****	***	ns
wt vs. G12D	ns	ns	ns	ns	ns	ns	ns
wt vs. G12V	ns	ns	ns	ns	ns	ns	ns
G12C vs. G12D	ns	ns	***	****	****	***	ns
G12C vs. G12V	ns	ns	****	****	***	****	ns
G12D vs. G12V	ns	ns	ns	ns	*	ns	ns

Statistical analysis was performed using two-way ANOVA test and Bonferroni post-test for multiple comparisons.

Figure 6b–d: BER pathway components. Growth inhibition assay.

	Cisplatin uM				
	0,625	1,5	2,5	5	10
wt vs. G12C	ns	**	*	***	ns
wt vs. G12D	ns	ns	ns	ns	ns
wt vs. G12V	ns	ns	ns	ns	ns
G12C vs. G12D	ns	****	*	****	ns
G12C vs. G12V	ns	****	****	****	ns
G12D vs. G12V	ns	ns	ns	ns	ns

Statistical analysis was performed using two-way ANOVA test and Bonferroni post-test for multiple comparisons.

	Cisplatin uM + MA				
	0,625	1,5	2,5	5	10
wt vs. G12C	ns	ns	ns	ns	ns
wt vs. G12D	ns	*	ns	ns	ns
wt vs. G12V	ns	*	ns	ns	ns
G12C vs. G12D	ns	**	ns	ns	ns
G12C vs. G12V	ns	**	*	*	ns
G12D vs. G12V	ns	ns	ns	ns	ns

Statistical analysis was performed using two-way ANOVA test and Bonferroni post-test for multiple comparisons.

	Cisplatin uM + PA				
	0,625	1,5	2,5	5	10
wt vs. G12C	ns	ns	ns	ns	ns
wt vs. G12D	ns	ns	ns	ns	ns
wt vs. G12V	ns	ns	ns	ns	ns
G12C vs. G12D	ns	ns	ns	ns	ns
G12C vs. G12V	ns	ns	ns	ns	ns
G12D vs. G12V	ns	ns	ns	ns	ns

Statistical analysis was performed using two-way ANOVA test and Bonferroni post-test for multiple comparisons.

Figure 7a: Caspase 3 and 7 activation: wild-type and mutant KRAS clones were treated with cisplatin, PA or MA (alone or in combination with cisplatin).

	wt	G12C	G12D	G12V
Ctr vs. cisplatin	****	ns	****	****
Ctr vs. PA	ns	ns	ns	ns
Ctr vs. PA+cisplatin	****	****	****	****
Ctr vs. METO	ns	ns	ns	ns
Ctr vs. METO+cisplatin	*	*	*	ns

Statistical analysis was performed using two-way ANOVA test and Bonferroni post-test for multiple comparisons.

Supplementary Figure S1b: Growth inhibition assay: response of cells to melphalan treatment detected by MTS assay.

	Melphalan uM							
	2.5	10	20	30	40	60	80	100
wt vs. G12C	ns	ns	ns	ns	ns	ns	ns	ns
wt vs. G12D	ns	ns	ns	ns	ns	ns	ns	ns
wt vs. G12V	ns	**	***	*	ns	ns	ns	ns
G12C vs. G12D	ns	ns	ns	ns	ns	ns	ns	ns
G12C vs. G12V	ns	ns	ns	ns	ns	ns	ns	ns
G12D vs. G12V	ns	ns	**	ns	ns	ns	ns	ns

Statistical analysis was performed using two-way ANOVA test and Bonferroni post-test for multiple comparisons.

Supplementary Figure S1c: Graphical presentation of ATM phosphorylation.

	Time after treatment start			
	2 h	16 h	24 h	48 h
wt vs. G12C	ns	****	*	**
wt vs. G12D	ns	ns	*	ns
wt vs. G12V	ns	ns	ns	ns
G12C vs. G12D	ns	***	****	***
G12C vs. G12V	ns	****	**	ns
G12D vs. G12V	ns	*	ns	*

Statistical analysis was performed using two-way ANOVA test and Bonferroni post-test for multiple comparisons.

Supplementary Figure S3c: Growth inhibition assay: response of cells to X-ray treatment.

	X-Ray Gy				
	0.5	1	2.5	5	10
wt vs. G12C	ns	ns	ns	ns	ns
wt vs. G12D	ns	ns	*	**	ns
wt vs. G12V	ns	ns	ns	ns	ns
G12C vs. G12D	ns	ns	ns	ns	ns
G12C vs. G12V	ns	ns	ns	ns	ns
G12D vs. G12V	ns	ns	ns	ns	ns

Statistical analysis was performed using two-way ANOVA test and Bonferroni post-test for multiple comparisons.

Supplementary Figure S4c: POLβ quantification.

	POLB
wt vs. G12C	***
wt vs. G12V	ns
wt vs. G12D	ns
G12C vs. G12V	*
G12C vs. G12D	***
G12V vs. G12D	ns

Statistical analysis was performed using one-way ANOVA test and Bonferroni post-test for multiple comparisons.

Figure S4f: Graphical presentation of ATM phosphorylation.

	wt	G12C	G12D	G12V
Ctr vs. cisplatin	**	ns	**	**
Ctr vs. PA	ns	ns	ns	ns
Ctr vs. PA+cisplatin	**	**	**	**
Ctr vs. METO	ns	ns	ns	ns
Ctr vs. METO+cisplatin	**	**	**	**

Statistical analysis was performed using two-way ANOVA test and Bonferroni post-test for multiple comparisons.

Supplementary Figure S5a: Growth inhibition assay: response of cells to MMS treatment detected by MTS assay.

	MMS uM						
	0.05	0.1	0.25	0.5	0.75	1	1.5
wt vs. G12C cl.2	ns	ns	**	**	**	*	ns
wt vs. G12C cl.4	ns	ns	***	***	***	*	ns
G12C cl.2 vs. G12C cl.4	ns	*	ns	ns	ns	ns	ns

Statistical analysis was performed using two-way ANOVA test and Bonferroni post-test for multiple comparisons.

Supplementary Figure S5g: Caspase 3 and 7 activation: KRAS(G12C) clone was treated with cisplatin, pamoic acid or meyhoxyamine (alone or in combination with cisplatin).

	G12C cl.4
Ctr vs. DDP	ns
Ctr vs. PA	ns
Ctr vs. PA_DDP	****
Ctr vs. METO	ns
Ctr vs. METO_DDP	ns

Statistical analysis was performed using two-way ANOVA test and Bonferroni post-test for multiple comparisons.

Supplementary Figure S5h: Growth inhibition assay: response of cells to cisplatin or cisplatin plus pamoic acid treatment detected by MTS assay.

	Cisplatin uM				
	1.5	2.5	5	7.5	10
wt cl.10 vs. G12C cl.18	***	ns	ns	***	***
wt cl.10 vs. G12D cl.11	*	ns	**	***	***
wt cl.10 vs. G12V cl.31	****	****	****	****	****
G12C cl.18 vs. G12D cl.11	****	***	****	****	****
G12C cl.18 vs. G12V cl.31	****	****	****	****	****
G12D cl.11 vs. G12V cl.31	ns	**	***	ns	ns

Statistical analysis was performed using two-way ANOVA test and Bonferroni post-test for multiple comparisons.

	Cisplatin uM + Pamoic Acid				
	1.5	2.5	5	7.5	10
wt cl.10 vs. G12C cl.18	ns	ns	***	****	****
wt cl.10 vs. G12D cl.11	**	*	***	****	***
wt cl.10 vs. G12V cl.31	**	****	****	****	***
G12C cl.18 vs. G12D cl.11	ns	ns	ns	ns	ns
G12C cl.18 vs. G12V cl.31	ns	**	ns	ns	ns
G12D cl.11 vs. G12V cl.31	ns	ns	ns	ns	ns

Statistical analysis was performed using two-way ANOVA test and Bonferroni post-test for multiple comparisons.

Supplementary Table S2: Percentage of cells in the cell cycle phase analysis presented in Figure 1E

	KRAS(wt)			KRAS(G12C)			KRAS(G12D)			KRAS(G12V)		
	G_1	S	G_2/M	G_1	S	G_2/M	G_1	S	G_2/M	G_1	S	G_2/M
Ctrl 24 h	34.7	49.5	15.8	31.4	53.8	14.8	34.5	50.7	14.4	33.3	50.4	16.3
Ctrl 48 h	51.6	37.8	10.6	66.6	25.2	8.2	50.7	40.1	9.2	52.4	36.8	10.8
24 h after treatment	12.9	47.2	39.9	33.4	47.1	19.5	19.0	51.2	29.8	17.3	51.2	31.6
48 h after treatment	31.5	41.4	27.1	52.0	35.6	12.4	36.7	45.3	18.0	34.7	44.9	20.4
